# Preclinical model for evaluating human TCRs against chimeric syngeneic tumors

**DOI:** 10.1136/jitc-2024-009504

**Published:** 2024-12-22

**Authors:** Aikaterini Semilietof, Evangelos Stefanidis, Elise Gray-Gaillard, Julien Pujol, Alessia D'Esposito, Patrick Reichenbach, Philippe Guillaume, Vincent Zoete, Melita Irving, Olivier Michielin

**Affiliations:** 1 Swiss Institute of Bioinformatics, Lausanne, Switzerland; 2 Department of Oncology, Ludwig Institute for Cancer Research Lausanne, University of Lausanne, Epalinges, Switzerland; 3 Precision Oncology, University Hospital of Geneva, Geneva, Switzerland

**Keywords:** Immunotherapy, Adoptive cell therapy - ACT, T cell, T cell Receptor - TCR, Tumor microenvironment - TME

## Abstract

**Background:**

The adoptive cell transfer (ACT) of T cell receptor (TCR)-engineered T cells targeting the HLA-A2-restricted epitope NY-ESO-1_157-165_ (A2/NY) has yielded important clinical responses against several cancers. A variety of approaches are being taken to augment tumor control by ACT including TCR affinity-optimization and T-cell coengineering strategies to address the suppressive tumor microenvironment (TME). Most TCRs of clinical interest are evaluated in immunocompromised mice to enable human T-cell engraftment and do not recapitulate the dynamic interplay that occurs with endogenous immunity in a treated patient. A variety of humanized mouse models have been described but they have limitations in immune reconstitution and are technically challenging to implement. Here, we have developed a chimeric syngeneic tumor model in which A2Kb transgenic C57BL/6 mice are engrafted with B16 expressing A2Kb:NY as a single chain trimer (SCT) and treated by ACT with murine T cells expressing A2/NY TCRs comprising human variable fused to mouse constant regions.

**Methods:**

We compared the function of a supraphysiological affinity A2/NY TCR (wtc51m), a computationally designed TCR in an optimal affinity range (DMβ), and a near non-binding TCR (V49I), engineered in both primary human and murine T cells by lentiviral and retroviral transduction, respectively. We evaluated a variety of strategies to stably express A2Kb:NY on the surface of mouse tumor cell lines including B16 melanoma, ultimately achieving success with an SCT comprising human β2m fused by GS linkers to both the NY-peptide and to α1 of the HLA complex. ACT studies were performed in B16-A2Kb:NY tumor-bearing, non-preconditioned immune-competent HLA-A*0201/H-2Kb (A2Kb) transgenic C57BL/6 mice and tumors characterized post-transfer.

**Results:**

We observed significantly improved function of DMβ-T cells as well as superior infiltration and tumor control upon ACT as compared to the control TCR-T cells. Moreover, with our chimeric syngeneic tumor model, we were able to track dynamic and favorable changes in the TME upon DMβ-T cell transfer.

**Conclusions:**

We have developed a robust, simple, and inexpensive preclinical strategy for evaluating human TCRs in the context of a fully competent murine immune system that can aid in the development of coengineered TCR-T cells and combination treatments translated to the clinic.

**WHAT IS ALREADY KNOWN ON THIS TOPIC**

- The adoptive transfer of T cell receptor (TCR)-engineered T cells has conferred important clinical responses against some solid cancers. Although affinity-optimized TCRs can augment T-cell function, the suppressive tumor microenvironment (TME) must be tackled and endogenous immunity harnessed to meaningfully improve tumor control by adoptive cell transfer (ACT).

**WHAT THIS STUDY ADDS**

- Most preclinical TCR-T cell studies comprise ACT of engineered human T cells against xenograft tumor models in immunocompromised mice and as such the full impact of endogenous immunity is not addressed. Here, we have established a preclinical tumor model allowing the testing of human TCRs of clinical interest against syngeneic tumors gene-modified to present the target HLAp and engrafted in A2Kb transgenic C57BL/6 mice.

**HOW THIS STUDY MIGHT AFFECT RESEARCH, PRACTICE OR POLICY**

- Our preclinical tumor model enables comprehensive testing of TCRs of clinical interest along with T-cell coengineering or combinatorial treatment strategies for reprogramming the TME and improving patient responses.

## Introduction

 Tumors are frequently infiltrated by T lymphocytes (ie, tumor infiltrating T lymphocytes, TILs), and while many are nonreactive bystanders,^1^ a proportion express T cell receptors (TCRs) specific for different types of tumor antigens, including cancer germline and tissue differentiation antigens, antigens associated with transforming oncoviruses, and mutation-derived neoantigens.^2^,^4^ The adoptive transfer of ex vivo expanded TILs has demonstrated important clinical responses for a variety of cancers including melanoma,^5^,^8^ breast,^9^ lung,^10^ epithelial,^11^ ovarian,^12^ and cervical,^13^ thus underlying a central role for T cells in tumor immunity. Notably, in February 2024, the Food and Drug Administration (FDA) approved a TIL product for advanced melanoma, Lifileucel (Amtagvi), the first cellular therapy approved for a non-hematological solid tumor-type.^14^ However, TIL therapy faces important challenges, including that tumor-derived T cells can be in a highly exhausted/suppressed state and difficult to expand, or not present if the tumor is cold.^15^ Moreover, TCRs targeting ‘self’ tumor antigens may be of suboptimal affinity due to thymic negative selection.^16^

To overcome these obstacles to TIL therapy, important research efforts have also been ongoing in the development of TCR-engineered T cells^17^ and in August 2024 the FDA approved a MAGE-A4-targeted TCR-T cell therapy for unresectable or metastatic synovial sarcoma, Afamitresgene Autoleucel (Tecelra), the first approved TCR gene therapy for a solid cancer.^18 19^ Nowadays, TCRs can be readily and stably introduced into T cells by a variety of viral^20^ and non-viral methods.^21^ Advantages are that TCRs having optimal binding properties can be preselected against defined targets and introduced into fit peripheral blood T cells, and potentially one day into universal donor T cells for off-the-shelf adoptive cell transfer (ACT).^22^

Briefly, TCRs are heterodimeric receptors comprising an α-chain and β-chain, each made up of a variable region and a membrane-proximal constant region. TCRs engage proteolytically derived antigenic peptides (p) presented on the target cell surface by human leucocyte antigen molecules (HLA), also known as the major histocompatibility complex (MHC). TCR contact with HLAp is largely mediated by the three complementary determining regions (CDRs 1–3) of the α-chain and β-chain variable regions. A productive TCR/HLAp encounter triggers a cascade of signaling events via the CD3 complex, culminating in different effector functions including IFN-γ production, proliferation, and target-cell killing by CD8^+^ T cells, for example.^23 24^

A variety of TCR specificities have been tested in the clinic for ACT of solid tumors.^25^ Of particular interest are TCRs targeting the cancer testis (CT) antigen NY-ESO-1 which is expressed by a broad range of cancers, including melanoma, sarcoma, and epithelial ovarian cancer,^26^,^28^ but in healthy adult tissues is restricted to male germ cells.^29^ HLA-A2/NY-ESO-1_157-165_ (A2/NY)-specific TCRs, including ones that have been affinity-enhanced, have shown important promise in the clinic, conferring a 50% overall response rate in metastatic synovial sarcoma, typically an immune desert.^30^ Previously, by structure-based computational design^31 32^ of a natural A2/NY TCR (BC1) near-identical in sequence to the well-characterized 1G4 TCR,^33^ we developed a panel of increasing affinity TCRs. We observed maximum in vitro function for T cells gene-modified to express TCR variants in the upper range of natural affinity (~5–1 µM).^34 35^ One of the TCRs, double mutant-β (DMβ; K_D_=1.91 µM) comprising amino acid replacements G50A and A51E in CDR2β has shown robust tumor control in xenograft models^36^ and favorable responses in the clinic.^37^

Along with receptor affinity and choice of target tumor antigen, it is now widely held that the immunosuppressive tumor microenvironment (TME) must also be addressed in order to improve clinical responses to T-cell therapies. This can be done by rational combinatorial treatments such as low-dose irradiation and immune checkpoint blockade,^38 39^ or T-cell coengineering like the enforced secretion of cytokines or decoy molecules to exploit endogenous immunity and support the transferred T cells.^20 25 40 41^ Robust preclinical testing of cancer immunotherapies is critical for advancing safe and effective approaches to the clinic. However, TCRs of translational interest are largely tested in the context of xenograft tumor models and an important shortcoming is the lack of most endogenous immune cells in immune-deficient mice (eg, NOD scid gamma; NSG) which can impact treatment outcome. For example, whereas regulatory T cells (Tregs) can constrain responses to ACT, endogenous natural killer (NK) cells, M1-like macrophages, and cytolytic T cells can be harnessed to augment tumor control.^40 41^

Here, with the aim of facilitating the development of ACT therapies with clinically relevant TCRs, we present a chimeric syngeneic tumor model in which murine T cells are retrovirally transduced^40^ to express hybrid TCRs (human variable fused to mouse constant regions) and adoptively transferred into non-preconditioned HLA-A2/H2Kb transgenic C57BL/6 mice engrafted with B16 melanoma gene-modified to cell-surface express a chimeric A2Kb:NY complex in the form of a single chain trimer (SCT). ACT of DMβ TCR-T cells significantly controlled tumors and reprogrammed the TME as compared with the control TCR-T cells. We conclude that our chimeric syngeneic tumor mouse model is a valuable tool for exploring combination treatments and coengineered TCR-T cells of interest for clinical translation.

## Results

### Hybrid A2/NY-targeted DMβ TCR comprising murine constant regions is functional in engineered human T cells

**Figure 1:**
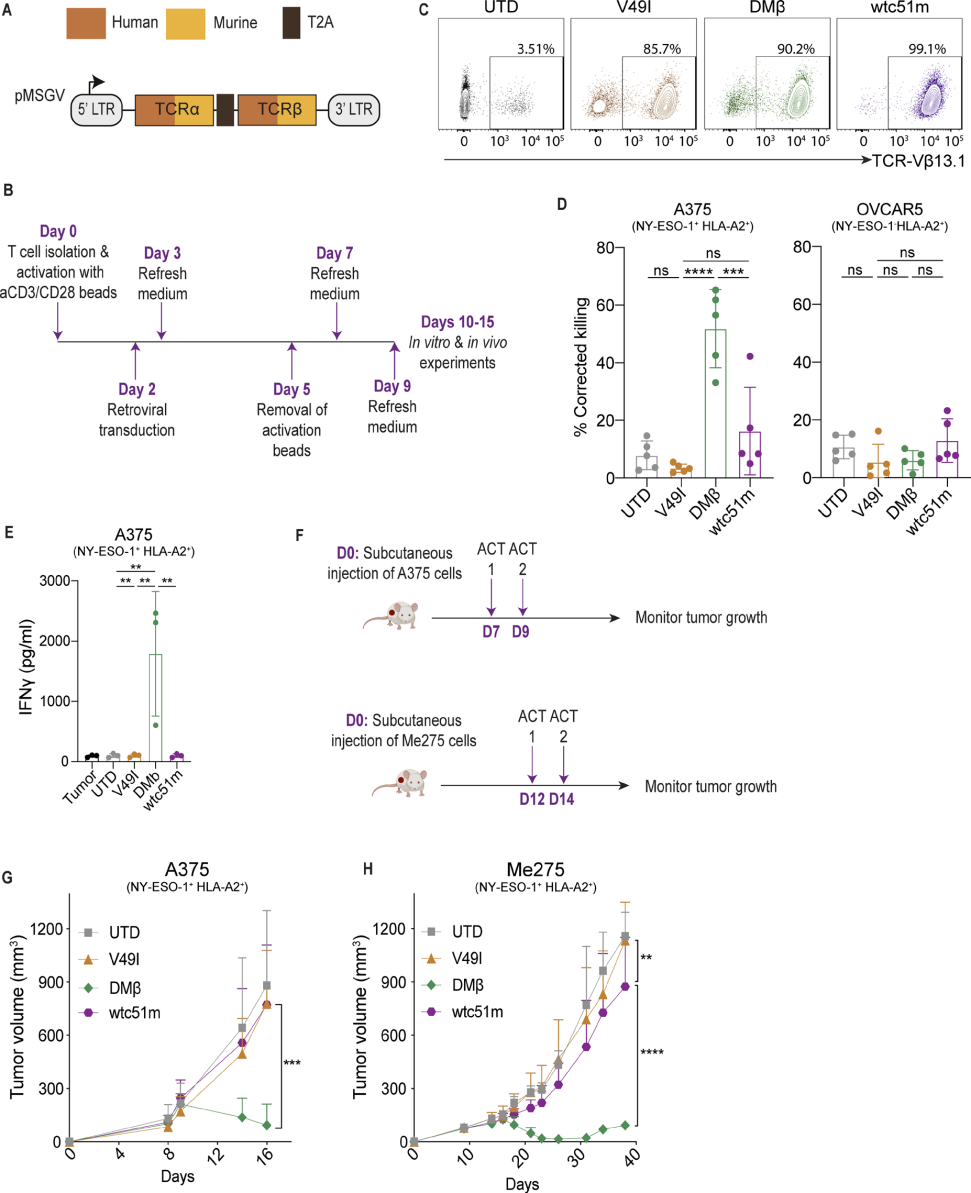
Primary human CD8^+^T cells transduced to express different-affinity hybrid A2/NY-TCRs exhibit expected relative function both in vitro and in vivo. (**A**) Schematic of retroviral constructs expressing hybrid TCRs comprising the human variable-regions α23.1 and β13.1 of the A2/NY TCRs (in orange), directly fused to murine constant regions (in yellow). (**B**) Primary human CD8^+^ T-cell activation, transduction, expansion, and culture conditions. (**C**) Transduction efficiency of CD8^+^ T cells with TCRs evaluated by flow cytometry staining with an anti-Vβ13.1 antibody (representative data of n=5 donors). (**D**) Frequency of Annexin V^+^ DAPI^+^ tumor cells in 24 hours co-cultures of TCR-T cells with either A375 or OVCAR5 tumor cells at an Effector:Target (**E:T**) ratio of 1:2. Frequency was corrected to tumor alone to account for spontaneous tumor cell death (n=5 donors). (**E**) IFNγ secretion levels by TCR-T cells on 24 hours co-culture with A375 tumor cells at E:T=1:2 (n=3 donors). (**F**) Schematic of ACT studies. (**G**) Control of subcutaneous A375 tumors in NSG mice post-ACT (n≥6 mice/group, data representative of 2 independent experiments). (**H**) Control of subcutaneous Me275 melanoma tumors in NSG mice post-ACT (n≥6 mice/group, data representative of two independent experiments). Statistical analysis was performed by one-way analysis of variance (ANOVA) (**D, E**), two-way ANOVA (**G, H**), with correction for multiple comparisons by post hoc Tukey’s test (**D, E, G,H**). **p<0.01, ***p<0.001, ****p<0.0001. ns, not significant; ACT, adoptive cell transfer; UTD, untransduced.

Previous studies have demonstrated that hybrid TCRs, comprising human variable regions fused to murine constant regions, are functional in engineered primary human T cells. This strategy has been devised to reduce α-chain and β-chain mispairing among the introduced and endogenous (native) TCRs.^42^ With the intent to transduce murine T cells with TCRs targeting A2/NY for ACT purposes, we built retroviral vectors encoding the variable regions of the affinity-optimized DMβ-TCR (K_D_=1.9 µM)^35^ fused to murine α-chain and β-chain constant-regions (figure 1A). Here, we set on a hybrid TCR design to minimize the potential impact on CD3 complex association and cellular activation of mouse T cells. As controls, we also built vectors to express a previously designed, near non-binding TCR variant V49I (amino acid replacement in CDR2β), and a supraphysiologic affinity TCR, wtc51m (G50A+A51I+G52Q+I53T in CDR2β; K_D_=0.015 µM), both in hybrid format. We previously showed little to no in vitro activity of V49I-T cells, and attenuated function for wtc51m-T cells,^34 35 43^ presumably in part due to impaired serial triggering.^44^

Primary human CD8^+^ T cells were purified and then retrovirally transduced and expanded (figure 1B), yielding similar high cell-surface expression levels of the 3 different hybrid TCRs (figure 1C). Co-culture of hybrid TCR-T cells with the A2^+^/NY^+^ melanoma cell line A375 revealed significantly higher cytotoxicity by DMβ-T cells than wtc51m-T cells, in line with our previous characterization of their fully human TCR counterparts.^35^ There was no killing of A375 cells by V49I-T cells (figure 1D), nor was there reactivity of any of the TCR-T cells against the A2^+^/NY^−^ ovarian cancer cell line OVCAR5. In addition to higher cytotoxicity, DMβ-T cells also secreted significantly more IFN-γ 24 hours post target-cell co-culture initiation than the other hybrid TCR-T cells (figure 1E).

Subsequently, we performed ACT studies in NSG mice bearing either A375 or Me275 A2^+^/NY^+^ subcutaneous melanoma tumors (figure 1F). For both models, DMβ-T cells significantly reduced tumor burden as compared with wtc51m-T cells, V49I-T cells and untransduced (UTD)-T cells (figure 1G,H), with wtc51m-T cells enabling marginal deceleration of Me275 tumor growth (figure 1H). Taken together, these data validate the expected relative functionality of the different-affinity hybrid A2/NY TCRs in human T cells; V49I is too weak, wtc51m too strong, and DMβ is in an optimal affinity range.

### Hybrid DMβ-TCR-engineered mouse T cells are functional both in vitro and in vivo

**Figure 2:**
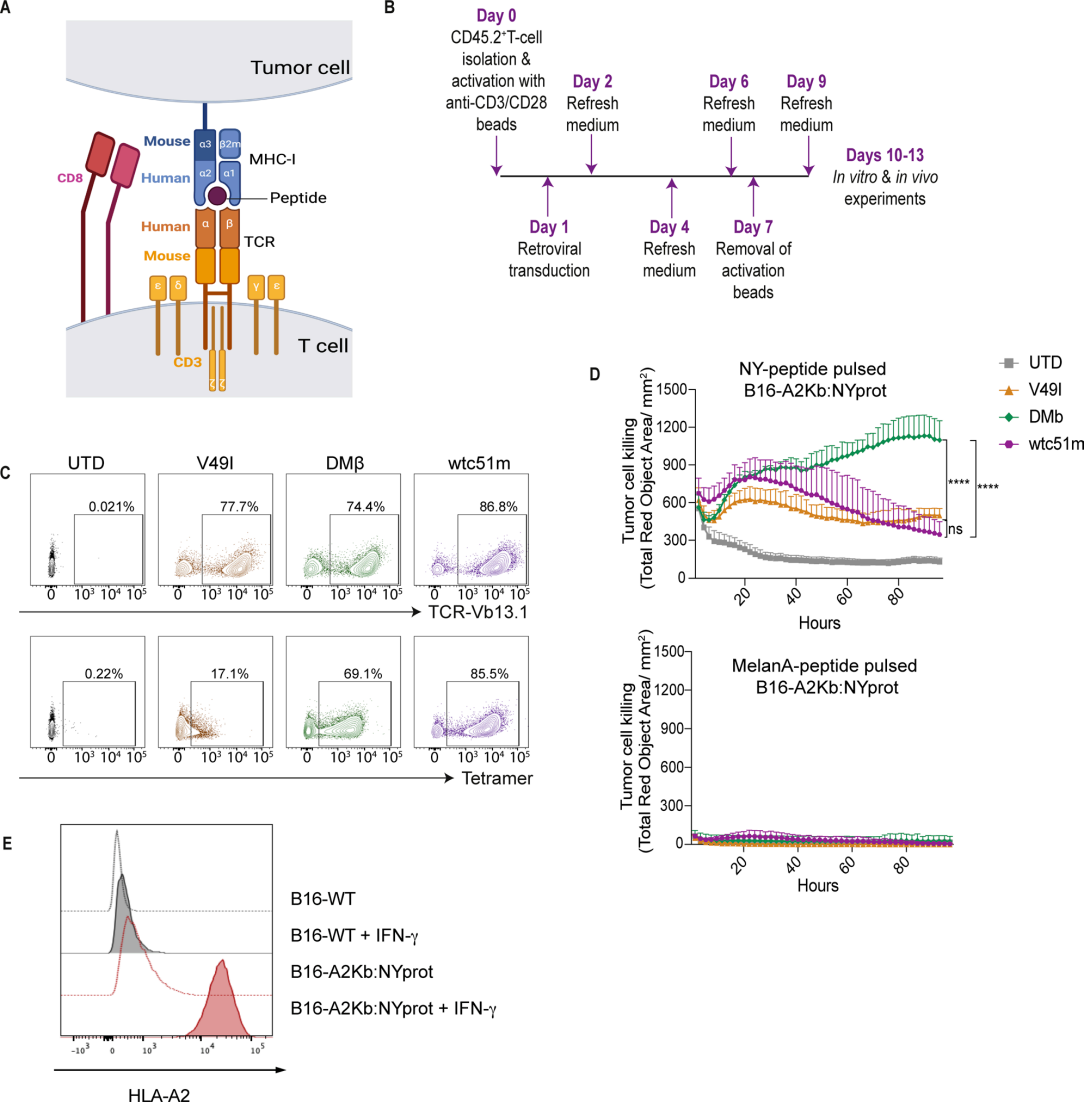
Hybrid DMβ-TCR redirects HLA-A2 transgenic mouse T cells against NY-peptide pulsed B16-A2Kb:NYprot melanoma target cells. (**A**) Schematic of a hybrid A2/NY TCR expressed by an HLA-A2 transgenic murine T cell engaging NY-peptide pulsed B16 tumor cells gene-modified to express a chimeric HLA-A2 molecule comprising α3 of H-2Kb to facilitate mouse CD8-coreceptor engagement. These cells also express the full NY protein but it is not processed and presented. (**B**) Schematic of primary mouse CD8^+^ T-cell activation, transduction, and expansion. (**C**) Transduction efficiency of mouse CD8^+^ T cells with hybrid TCRs evaluated by an anti-Vβ13.1 antibody staining (top) and specific tetramer (bottom), (representative data of n>5 donors). (**D**) IncuCyte live-cell imaging to evaluate killing by hybrid TCR-T cells at an E:T ratio of 2:1 of the B16-A2Kb:NYprot cell line peptide-loaded with 10 µM of either NY-peptide or MelanA-peptide. Cytotox red is added in the co-culture to visualize and quantify target cell death (n=3 donors). (**E**) Flow cytometric analysis of B16 and B16-A2Kb:NYprot cells co-cultured or not in the presence of IFN-γ overnight and cell-surface stained for HLA-A2. Statistical analysis by two-way ANOVA (**D**), with correction for multiple comparisons by post hoc Tukey’s test. ****p<0.0001. ns, not significant; ANOVA, analysis of variance; E:T, Effector:Target; MHC, major histocompatibility complex; UTD, untransduced.

We next sought to engineer mouse T cells derived from the spleens of HLA-A*0201 /H-2Kb (A2Kb) transgenic C57BL/6 mice with the hybrid A2/NY TCRs and evaluate their in vitro function (figure 2A). The A2Kb mice, previously generated by Vitiello *et al*,^45^ and validated by others,^46^ express a chimeric human-mouse HLA class I complex in which the α1-domains and α2-domains comprising the peptide-binding groove are of human origin, and α3 is murine to enable murine CD8 coreceptor engagement.

With our optimized retrovirus transduction and T-cell expansion protocol (figure 2B) described in Lanitis *et al*,^40^ we achieved similar high cell-surface expression of the DMβ and wtc51m TCRs in splenic mouse A2Kb T cells as assessed by tetramer staining (figure 2C, lower panel). As expected, we did not detect tetramer binding by the V49I-T cells and as such anti-TCR Vβ13.1 antibody (Ab) was used to evaluate transduction efficiency in all subsequent experiments (figure 2C, upper panel). To assess the functionality of the hybrid TCR-T cells in vitro, by retroviral transduction and selection we generated a B16 melanoma cell line stably expressing a chimeric A2Kb complex (human α1-α2 peptide-binding groove and mouse α3) along with the full NY-ESO protein (B16-A2Kb:NYprot; figure 2A). IncuCyte live-cell imaging of co-cultures of B16-A2Kb:NYprot tumor cells revealed robust killing by mouse DMβ-T cells on pulsing with NY-peptide (SLLMWITQA; C9A avoids unwanted disulfide bridge formation) (figure 2D, top) but not if pulsed with a non-specific HLA-A2 restricted MelanA_26-35_-peptide (ELAGIGILTV; replacement E2L increases binding stability of the peptide)^47^ (figure 2D, bottom). These co-culture assays were performed following B16-A2Kb:NYprot exposure to IFN-γ which upregulated A2Kb on their cell-surface (figure 2E).

### Murine tumor cell lines can be gene-modified to cell-surface express Chimeric A2Kb:NY as an SCT

In order to perform in vivo studies, we next needed to gene-modify murine tumor cells to stably cell-surface express the target HLAp complex. Indeed, we observed that in the absence of NY-peptide pulsing of the B16-A2Kb:NYprot tumor cells protein described above (online supplemental figure 1A, left top) there was no reactivity of DMβ-T cells (online supplemental figure 1A, right). Assuming proteasome targeting/degradation to be problematic, we further fused a murine ubiquitin tag (Ubi) to the NY-ESO-1 protein, B16-A2Kb:UbiNYprot, (online supplemental figure 1A, left bottom)^48^,^50^ or the NY peptide directly (not shown) but DMβ-T cells remained non-responsive (online supplemental figure 1A, right).

We subsequently moved onto an SCT format to circumvent the need for peptide processing and loading onto A2Kb in the murine tumor cells. Briefly, murine β2m was fused by flexible GS linkers^51^ to both the NY-peptide and A2Kb further harboring the amino acid replacement Y84A (B16-A2Kb:NY-SCT-Y84A) in α1 previously described to stabilize peptide binding (online supplemental figure 1B, left top). In addition, we tested a non-native disulfide bridge (db; L2C and Y84C; B16-A2Kb:NY-dbSCT) described to improve peptide stability^52 53^ (online supplemental figure 1B, left bottom) but DMβ-T cells were not reactive against either (online supplemental figure 1B, right). We questioned if β2m of murine origin could be destabilizing to the chimeric A2Kb complex, or if the introduced amino acid replacements (Y84A or L2C+Y84C) were problematic.^54^

**Figure 3:**
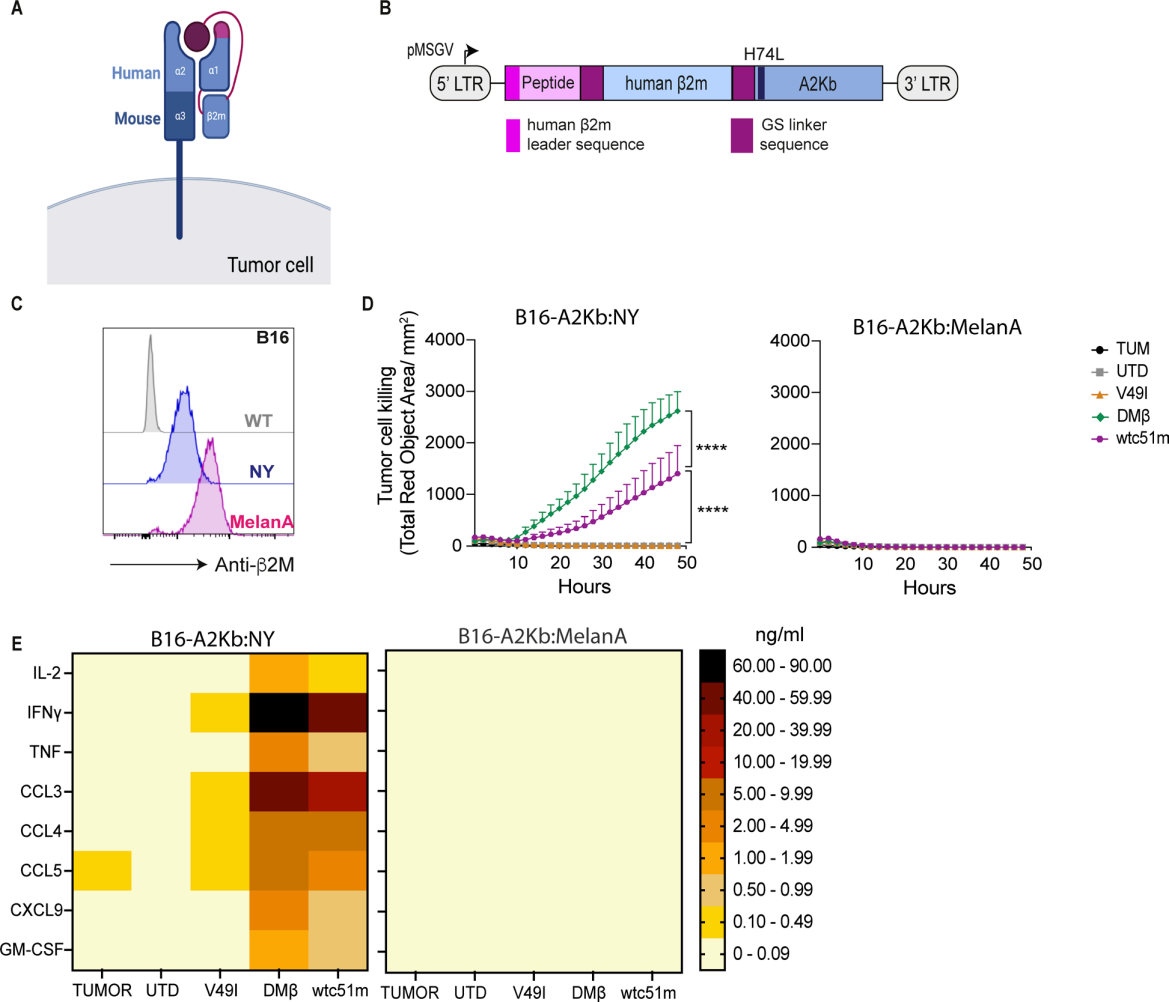
Hybrid A2/NY TCR-T cells are reactive against B16 tumor cells gene-modified to express a single chain trimer of A2Kb and the NY peptide. (**A**) Schematic of the functional single chain trimer (SCT) in which human β2m is connected by GS linkers to both human α1 and the NY-peptide. The H74L amino acid substitution is depicted in pink on the α1-chain. (**B**) Schematic of the retroviral vector encoding the SCT. (**C**) Anti-human β2m antibody staining of STC-engineered B16 tumor cells. (**D**) Cytotoxicity of B16-A2Kb:NY cells (left) by TCR-T cells at an Effector to Target (**E:T**) ratio of 2:1, followed by IncuCyte live cell imaging using Cytotox red dye (n=3 donors). B16-A2Kb:MelanA cells were used as a control (right). (**E**) Cytokine and chemokine secretion by TCR T cells post 24 hours co-culture with B16-A2Kb:NY (left) and B16-A2Kb:MelanA (right) tumor cells at E:T ratio=1:1 (n=3 donors) measured with CBA. Tumor cells alone were used as a control. Statistical analysis by two-way ANOVA (**D**), with correction for multiple comparisons by post hoc Tukey’s test. ****p<0.0001. ANOVA, analysis of variance; E:T, Effector:Target; UTD, untransduced.

We next came up with a plan of building an SCT comprising human β2m fused by GS linkers to both the peptide and to α1 comprising H74L described to stabilize peptide binding^55^ (figure 3A). Prior to experimental testing, we performed structure-based homology modeling of the TCR/SCT complex using the experimental structure of the 1G4 TCR bound to human A2/NY (PDB ID 2bnr) as a base.^33^ We observed that the GS linker between human β2m and α1 is long enough to connect them without perturbing the overall structure of the HLAp complex (online supplemental figure 2A). In addition, the GS linker connecting the NY peptide to β2m is sufficiently long and flexible to avoid steric hindrance with HLA and allow binding of the NY peptide in the HLA groove, similar to the experimental structure of the non-covalently bound peptide (online supplemental figure 2B). This GS linker is also distant from the TCR binding interface enabling the TCR to bind to the SCT in the same way as to the wild-type human A2/NY complex (online supplemental figure 2A,B).

Encouraged by the modeling, we proceeded to build retroviral constructs encoding this SCT (abbreviated A2Kb:NY; figure 3B) and transduced B16 melanoma cells (B16-A2Kb:NY). As a negative control, B16 cells were engineered with the same design SCT but comprising the MelanA/MART-1_26-35_ peptide (B16-A2Kb:MelanA). By anti-human β2m antibody staining we detected the complexes at the surface of the engineered B16 tumor cells (figure 3C). Moreover, in co-culture assays we observed significant specific killing of B16-A2Kb:NY cells but not B16-A2Kb:MelanA cells by murine DMβ-T cells (figure 3D), as well as cytokine secretion in the presence of the target tumor cells (figure 3E). We observed some reactivity of supraphysiological affinity wtc51m-T cells against B16-A2Kb:NY cells, possibly due to higher levels of the HLAp SCT than may be naturally present on tumor cells (ie, A375, Me275). This is in line with our previous in vitro characterization of fully human wtc51m-T cells showing that their activity can be augmented if target tumor cells are pulsed with higher levels of NY peptide^34^ which may help to compensate for impaired serial triggering by T cells expressing TCRs of too high affinity.

Lastly, we engineered MC38 cancer colon cells^56 57^ to express our functional SCT presenting the NY-peptide or MelanA-peptide (online supplemental figure 3A). DMβ-T cells demonstrated significantly higher reactivity against MC38-A2Kb:NY than V49I-T cells and wtc51m-T cells, and there was no reactivity of any of the A2/NY TCR-T cells against MC38-A2Kb:MelanA cells (online supplemental figure 3B–C). Higher levels of reactivity by V49I-T cells and wtc51m-T cells against MC38-A2Kb:NY (than B16-A2Kb:NY) may be due to higher expression levels of A2Kb:NY per cell, or/and lower inhibitory mechanisms at play, or/and higher susceptibility of MC38 to other forms of cell death (eg, via TNFα or FASL).

### Hybrid DMβ-T cells control B16-A2Kb:NY tumors and reshape the immune microenvironment

**Figure 4:**
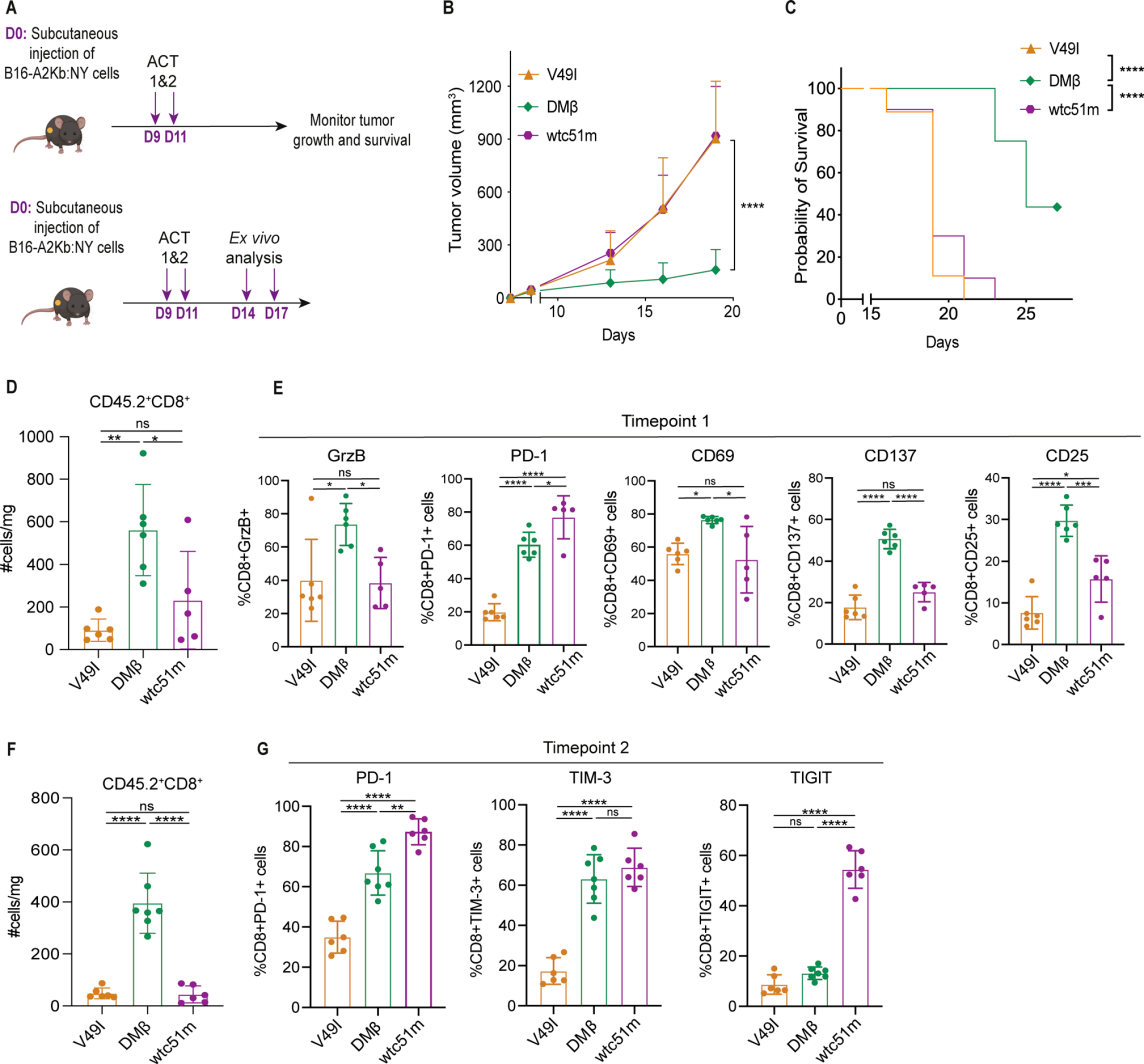
Hybrid DMβ-T cells exert control of B16-A2Kb:NY tumors and exhibit a superior fitness profile to V49I- and wtc51m-T cells post-transfer. (**A**) Schematic of ACT studies to evaluate tumor control (top) and immune cell infiltration over time (days; **D**) (bottom). (**B**) Control of subcutaneous B16-A2Kb:NY tumors in HLA-A2/H-2Kb C57BL/6 transgenic mice, following ACT of mouse CD45.2^+^CD8^+^ TCR-T cells (n≥7 mice/group). (**C**) Mouse survival post-ACT (n≥7 mice/group). (**D**) Number of intratumoral adoptively transferred CD45.2^+^CD8^+^ TCR T cells 3 days post-ACT (timepoint 1) (n≥5 mice/ group). (**E**) Phenotypic analysis of markers for function, inhibition, and activation of adoptively transferred intratumoral CD45.2^+^CD8^+^ TCR-T cells (n≥5 mice/ group), shown as percentage of the T cells. (**F**) Number of intratumoral adoptively transferred CD45.2^+^CD8^+^ TCR-T cells 6 days post-ACT (time point 2) (n=6 mice/ group). (**G**) Percentage expression of inhibitory cell-surface receptors by intratumoral CD45.2^+^CD8^+^ TCR-T cells 6 days post-ACT (n=6 mice/ group). (**B–G**) Representative data of two independent experiments. Statistical analysis by two-way ANOVA (**B**), Mantel-Cox (**C**) or one-way ANOVA (**D–G**), with correction for multiple comparisons by post hoc Tukey’s test (**B–G**). *p<0.05, **p<0.01, ***p<0.001, ****p<0.0001. ns, not significant, ACT, adoptive cell transfer; ANOVA, analysis of variance.

We achieved engraftment as well as similar growth curves for B16 wild-type, B16-A2Kb:NY and B16-A2Kb:MelanA in A2Kb transgenic C57BL/6 mice (online supplemental figure 4A),^46^ enabling us to move forward with comparative ACT studies. Briefly, we engineered CD45.2^+^ CD8^+^T cells from A2Kb C57Bl/6 mice with the different hybrid TCRs and transferred them into CD45.1^+^ A2Kb transgenic C57Bl/6 mice bearing subcutaneous B16-A2Kb:NY tumors (figure 4A). The mice were not preconditioned (ie, no full-body irradiation or chemotherapy) prior to transfer to maintain the endogenous immune compartment. The hybrid CD8^+^ DMβ-T cells efficiently controlled target tumors and prolonged survival (figure 4B,C) while V49I-T cells and wtc51m-T cells did not delay tumor outgrowth and treated mice had similar limited survival (figure 4B). Transfer of DMβ-T cells had no impact on tumors presenting A2Kb:MelanA (online supplemental figure 4B).

Ex vivo analysis at day-3 post-ACT (time point 1) revealed a significantly elevated presence in tumors of DMβ-T cells (figure 4D), further characterized by higher expression of Granzyme B and activation markers CD137, CD69, CD25, as compared with V49I-T cells and wtc51m-T cells, both in terms of percentage of T cells (figure 4E) and MFI (online supplemental figure 5A). We also observed that the supraphysiologic affinity wtc51m-T cells expressed significantly higher levels of the inhibitory maker PD-1, in line with previous in vitro findings^58^ (figure 4E and online supplemental figure 5A). At 6 days post-ACT (time point 2), we observed fewer infiltrated T lymphocytes, likely in part because the mice had not been pre-conditioned, but DMβ-T cells were present at significantly elevated levels as compared with V49I-T cells and wtc51m-T cells (figure 4F). At day-6 post-ACT, we also observed significantly higher upregulation of the inhibitory markers PD-1, TIGIT, and TIM-3 by the wtc51m-T cells, both with respect to percentage of T cells (figure 4G) and MFI (online supplemental figure 5B). Moreover, at day-6 post-ACT, wtc51m-T cells demonstrated the highest percentage of PD-1^+^TIM-3^+^ expression and of TIGIT^+^ in PD-1^+^TIM-3^+^ T expression (online supplemental figure 5C), indicating that they may be in a more exhausted state than DMβ-T cells.

**Figure 5:**
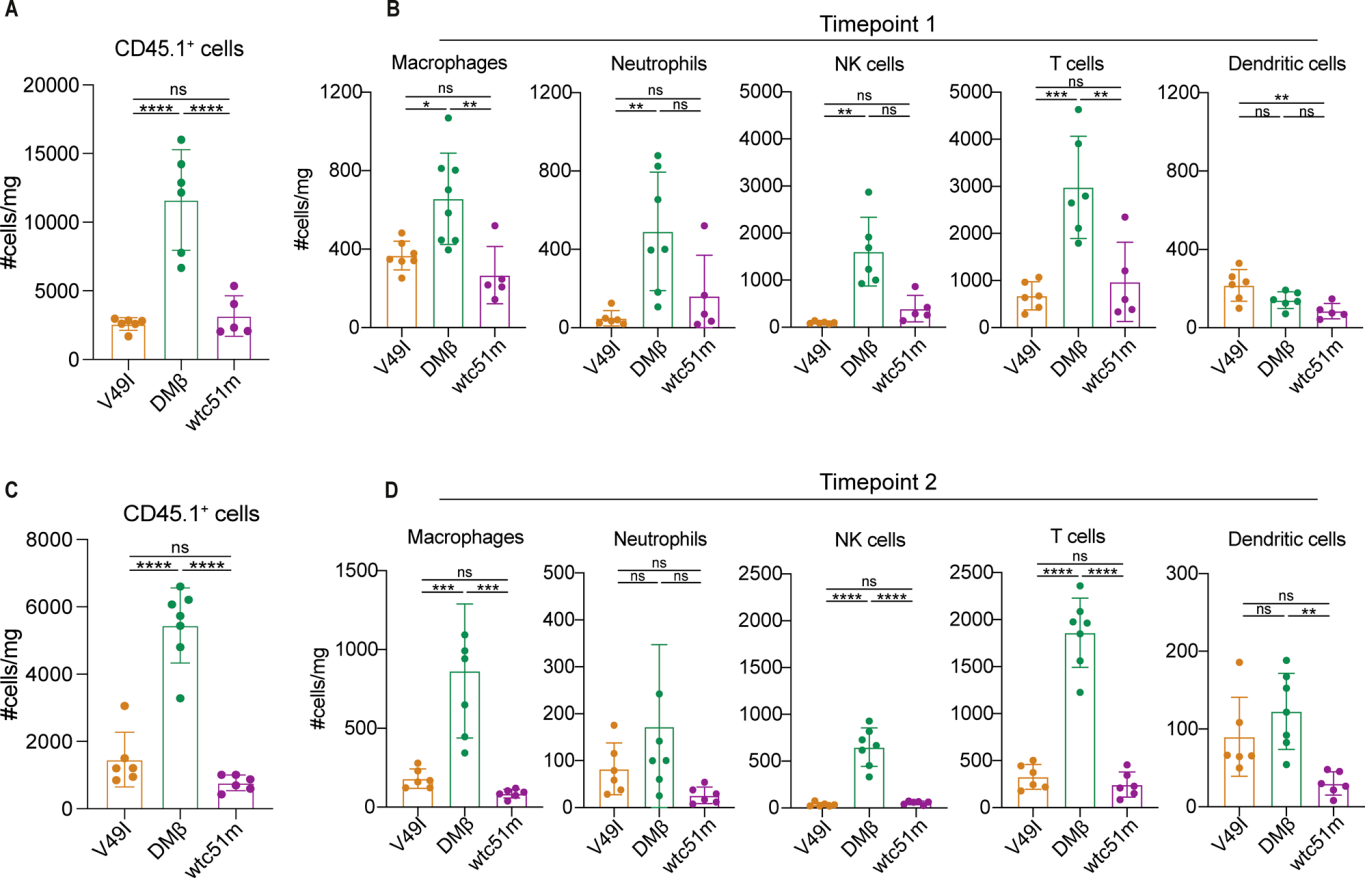
The adoptive transfer of hybrid DMβ-T cells is associated with elevated levels of endogenous tumor immune infiltrate. (**A**) Number of intratumoral endogenous immune CD45.1^+^ cells 3 days post-ACT (n≥5 mice/ group). (**B**) Number of intratumoral CD45.1^+^ macrophages, neutrophils, dendritic cells, T cells and NK cells 3 days post-ACT (n≥5 mice/ group). (**C**) Number of intratumoral endogenous immune CD45.1^+^ cells 6 days post-ACT (n=6 mice/ group). (**D**) Number of intratumoral CD45.1^+^ macrophages (CD3^-^CD19^-^Ly6C^-^MHC-II^+^CD11c^+^F4/80^+^CD64^+^), neutrophils (CD3^-^CD19^-^Ly6G^+^), dendritic cells (CD3^−^CD19^−^Ly6C^−^MHC-II^+^CD11c^+^F4/80-CD64^-^CD11b^+^), T cells (CD3^+^) and NK (CD3^−^CD19^−^CD161^+^) cells 6 days post-ACT (n=6 mice/ group). Statistical analysis by one-way ANOVA with correction for multiple comparisons by post hoc Tukey’s test (**A–D**). *p<0.05, **p<0.01, ***p<0.001, ****p<0.0001. Representative data of two independent experiments. ns, not significant ACT, adoptive cell transfer; ANOVA, analysis of variance; MHC, major histocompatibility complex.

Investigation of the endogenous immune system at day-3 post-ACT (time point 1) revealed a significantly higher overall immune infiltration/presence in the tumors of mice treated by DMβ-T cells (figure 5A), including of macrophages, neutrophils, NK cells, and T cells (figure 5B). Similar trends were observed at day-6 post-ACT (figure 5C, D). Notably, in mice treated by DMβ-T cells the endogenous T cells presented significantly higher intracellular Granzyme B levels (ie, indicating stronger effector function) and a trend for lower levels of inhibitory receptors PD-1 and TIM-3 (online supplemental figure 5D). Taken together, these data show that the higher infiltration and activity levels of DMβ-T cells post-ACT elevates the endogenous tumor immune microenvironment.

## Discussion

The adoptive transfer of gene-modified T-cells holds tremendous immunotherapeutic potential as exemplified by the curative responses now documented for some hematological cancer patients treated by chimeric antigen receptor (CAR)-T cells targeting the B-cell lineage antigen CD19.^59^ While TCR-engineered T cells may not yet have achieved such robust clinical outcomes, TCRs offer many advantages over CARs, including that they are not limited to targeting surface antigens, they confer exquisite sensitivity allowing responsiveness to very low levels of target HLAp,^60^ and they may more readily enable T-cell penetration into solid tumors. Hence, extensive research efforts are ongoing and warranted in the development of TCR therapeutics^17^ as well as of preclinical in vivo tools for comprehensively testing efficacy and safety.

Previously by computational design, we developed a panel of increasing-affinity TCRs targeting A2/NY and observed maximum in vitro function of T cells engineered to express ones in the upper range of natural affinity.^34 35^ We also demonstrated robust control of xenograft tumors in immunocompromised mice with affinity-optimized TCR-T cells as compared with wild-type TCR-T cells.^36 61^ However, binding-enhanced TCRs alone are insufficient to drive curative responses to ACT^62^,^64^ as the suppressive TME can impair their persistence and sustained function.^65^ Indeed, it is widely held that endogenous immunity must be exploited and adaptive and innate immune checkpoints overcome to augment solid tumor control by ACT.

We have previously demonstrated, for example, the use of low-dose irradiation to reverse immune desertification combined with immune checkpoint blockade, CD40 agonist antibody, and cyclophosphamide to reprogram endogenous adaptive and innate immunity for improved control of ovarian cancer (ID8).^38^ In addition, we have tested a range of T cell coengineering strategies for augmenting tumor control by ACT.^41 61 66 67^ For example, we showed that the enforced secretion of IL-15 by CAR-T cells in ACT studies against B16 melanoma not only improved T-cell fitness and function but was also associated with tumor immune microenvironment reshaping including NK cell activation and a reduced presence of M2-like macrophages.^40^

Although humanized models in which immune-compromised mice^68^ are engrafted with human fetal liver or adult CD34^+^ progenitor cells can enable testing of immunotherapies including autologous ACT,^69^,^71^ not all immune components, in particular myeloid and NK cells, are represented. The development of transgenic mice expressing various combinations of human cytokines has helped improve/expand reconstitution,^72^,^74^ but overall these strains and strategies are expensive and technically challenging to implement. Moreover, unless the progenitor cells and tumors are from the same donor, HLA mismatching can cause alloreactivity and influence tumor control.

With the ultimate goal of developing effective TCR-T cell therapies against solid tumors, here we set out to establish a simple chimeric syngeneic tumor model in order to test A2/NY TCRs of clinical interest in the context of a fully competent immune system in A2Kb transgenic C57BL/6 mice. We began by generating hybrid TCRs comprising human variable and mouse constant regions. We chose three A2/NY TCRs for our proof-of-principle study, near non-binding V49I, supraphysiological affinity wtc51m which attenuates T-cell function, and affinity-optimized DMβ.^35^ We observed the same relative function of the different-affinity hybrid TCRs engineered into both human and mouse T cells.

The gene-modification of mouse tumor cell lines to stably cell-surface express target HLAp proved to be challenging. For the complex, we fused the α1-α2 peptide binding groove of human origin to mouse α3-domain (ie, A2Kb) to enable mouse CD8 coreceptor engagement. Based on our data, we speculate that the mouse proteasome is incompatible with processing the human NY-ESO-1 protein and/or to enable NY-peptide loading onto chimeric A2Kb molecules. Ultimately, we developed a SCT including human β2m fused by GS linkers to both the NY-peptide and to α1 comprising H74L described to stabilize peptide binding.^55^ Future studies should compare DMβ-T cell reactivity against the SCT without the H74L replacement which may not be needed. Notably, structure-based homology modeling of the TCR/SCT complex using the experimental structure of the 1G4 TCR bound to human A2/NY (PDB ID 2bnr) as a base^33^ indicated no changes in peptide binding to the modified HLA groove or interference in TCR binding to the SCT as compared with the wild-type TCR:A2/NY complex.

Using B16 melanoma cells stably expressing the A2Kb:NY SCT we were able to perform ACT studies and showed significant tumor control and survival for tumor-bearing mice treated with DMβ-T cells. We also explored T-cell fitness post-transfer and the impact of ACT on endogenous immunity. At day-3 post-ACT, we observed significantly elevated levels of infiltrating DMβ-T cells characterized by higher expression of Granzyme B, CD137, CD69, and CD25 as compared with V49I-T cells and wtc51m-T cells. Notably, the supraphysiological affinity wtc51m-T cells expressed significantly higher levels of the inhibitory maker PD-1 at day 3, and of PD-1, TIGIT, and TIM-3 by day 6. Moreover, a higher percentage of wtc51m-T cells co-upregulated PD-1 and TIM-3 suggesting that they are in a more exhausted state^75^ than DMβ-T cells. At this second time point, there were also fewer transferred T cells in the tumors, likely because the mice were not preconditioned prior to ACT.

Future studies should explore combination therapies such as immune checkpoint blockade with the hybrid A2/NY-T cells, as well as coengineering strategies like enforced proinflammatory cytokine secretion (eg, IL-12, IL-18) to rationally exploit endogenous immunity.^25 41^ Also to be elucidated are the molecular mechanisms behind such vast differences in in vitro function and tumor control between the different-affinity A2/NY-T cells.^76^ Overall, we have presented a robust and straight-forward in vivo system for optimizing adoptive TCR-T cell therapy and facilitating the translation of more effective approaches that exploit endogenous immunity to the clinic.

## Materials and methods

### Mice

NOD.Cg-Prkdc^scid^ Il2rg^tm1Wjl^/SzJ (NSG) mice were purchased from the Jackson Laboratory and subsequently maintained and bred in-house under specific opportunist pathogen-free conditions at the Epalinges UNIL animal facility. CD45.1^+^ HLA-A2/H-2Kb and CD45.2^+^ HLA-A2/H-2Kb C57BL/6 transgenic mice were generated and bred in-house.^46^ All in vivo experiments were conducted in accordance with approved licenses from the Service of Consumer and Veterinary Affairs of the Canton of Vaud (VD3517 and VD3517×1). Each cage housed five mice and provided an enriched environment with unrestricted access to food and water. During in vivo studies, the mice were monitored at least every 2 days for signs of distress, and they were euthanized at endpoint by carbon dioxide overdose.

### Cell lines

Human embryonic kidney (HEK-293T), Phoenix-Ecotropic (ECO), human melanoma A375 (HLA-A*0201^+^NY-ESO-1^+^), mouse melanoma B16 (H-2Kb^+^) and mouse colon carcinoma MC38 (H-2Kb^+^) cell lines were purchased from the ATCC. Human melanoma Me275 cells (HLA-A*0201^+^, NY-ESO-1^+^) were kindly provided by Professor Daniel Speiser (University of Lausanne) and human ovarian cancer OVCAR5 cells were a gift from Professor Coukos (formerly at the University of Pennsylvania). A375 and OVCAR5 cell lines were engineered with NucLight Red lentivirus (IncuCyte) to stably express nuclear-restricted mKate2 fluorescent protein to track in vitro activity according to the manufacturer’s instructions. Various B16 and MC38 variants were generated by retrovirally transducing parental B16 and MC38 to stably express the corresponding chimeric proteins, as described below. Transduced cells were selected either by supplementation of the culture medium with antibiotics and/or by FACS sorting with antibodies against the expressed molecules. Human melanoma cells were maintained in IMDM (Thermo Fisher Scientific) supplemented with 10% FCS and 1% Penicillin/Streptomycin (P/S), mouse tumor cell lines were cultured in DMEM (Thermo Fisher Scientific) supplemented with 10% FCS and 1% P/S and the rest in RPMI-1640 Glutamax with 10% FCS and 1% P/S.

### Molecular cloning of TCRs

cDNA sequences of human near non-binding V49I, affinity-optimized DMβ and supraphysiological-affinity wtc51m TCR variants AV23.1 and BV13.1 genes, specific for NY-ESO-1_157-165_ epitope presented by HLA-A*0201, were derived from previous constructs in the lab.^35^ Chimeric human-mouse TCR variants were generated by replacing the human constant regions of all TCRs with the corresponding mouse sequences. The sequences were codon-optimized and synthesized by GeneArt (Thermo Fisher) in a single cassette separated by a 2A sequence self-cleaving peptide sequence. The retroviral vector pMSGV (murine stem cell virus (MSCV)-based splice-gag vector) comprising the MSCV LTR was used as the backbone for all TCR cassettes, which were subcloned into it using standard molecular biology techniques.

### Molecular cloning of A2Kb/peptide complexes

To generate B16-A2Kb:NY, B16-A2Kb:MelanA, MC38-A2Kb:NY and MC38-A2Kb:MelanA tumor cells, SCT constructs were built, comprising a human β2m leader sequence immediately fused to the NY-ESO-1_157-165_ or MelanA_26-35_ peptide, respectively, immediately connected to a GS-linker (G_4_S)_3_, immediately fused to mature human β2m and then linked by (G_4_S)_4_ to chimeric A2Kb (α1-α2 of human origin and α3 of mouse origin). A single amino acid substitution (H74L) was included in the α1-domain of the peptide docking groove to stabilize peptide binding.^55^ The SCT constructs were synthesized as a single cassette (GeneArt-ThermoFisher Scientific) and cloned into a blasticidin-resistant pMSGV retroviral vector. To generate B16-A2Kb:NYprot cells, the chimeric A2Kb sequence, and the full human NY-ESO-1 protein were synthesized in a single cassette, separated by a 2A sequence, and cloned in a blasticidin-resistant pMSGV retroviral vector. For B16-A2Kb:UbiNYprot, a mouse ubiquitin tag was added before the NY-ESO-1 sequence. The other 2 SCTs tested (that were not functional) included a mouse β2m leader sequence and mouse β2m along with a different stabilizing mutation in the peptide-binding groove (Y84A),^52^ or comprising a non-native disulfide bridge (L2C, Y84C) reported to increase peptide binding.^53 77^ All constructs were synthesized using GeneArt and cloned in a blasticidin-resistant pMSGV vector using standard molecular cloning techniques.

### Modeling the three-dimensional structure of the HLAp SCT and A2/NY TCR

The 3D structural model of the HLAp SCT and A2/NY (1G4) TCR complex was obtained by homology modeling using the Modeller program V.9.^78^ The HLAp SCT present in this model comprises human α1 and α2, murine α3, and human β2m. In addition, GS linkers were introduced between the NY-peptide and human β2m, as well as between β2m and α1. The experimental structure of the 1G4 TCR bound to the human A2/NY complex (PDB ID 2bnr^33^) was retrieved from the Protein Data Bank (PDB)^79^ and used as a template for the homology modeling. The sequence alignment between the TCR and the HLAp complex under investigation and the template was performed using MUSCLE.^80^ One thousand models were generated and scored according to the Modeller objective function. The best-rated model was finally selected and analyzed. Molecular graphics were produced using the UCSF Chimera software.^81^

### Production of retroviral particles

High-titer, replication-defective retrovirus was generated as previously described.^40 61^ Briefly, for the transfection of human cells with retroviral particles, HEK293T cells were co-transfected with 21 µg pMSGV transfer plasmid and 18 µg pMD22.-Gag/Pol and 7 µg pMD RD114 (feline endogenous virus envelope glycoprotein) retroviral packaging vectors, using a mix of OptiMEM medium (Thermo Fisher Scientific) and Turbofect (Thermo Fisher Scientific). To produce mouse ecotropic retroviral particles, Phoenix-ECO cells were co-transfected with 21 µg pMSGV transfer plasmid and 14 µg pCL-ECO retroviral packaging plasmid in the presence of OptiMEM and Turbofect. Culture supernatants were collected 48 hours or/and 72 hours post-transfection and concentrated by ultracentrifugation at 24,000×g for 2 hours. Concentrated virus was stored at −80°C until use.

### Human T-cell isolation, stimulation, viral transduction, and expansion

Healthy donor buffy coat products were purchased from the Transfusion Interrégionale CRS (Epalinges, Switzerland). PBMCs were isolated on day 0 using Lymphoprep (Axis-Shield) density gradient centrifugation and CD8^+^ T cells were purified using a CD8 negative selection kit (StemCell Technologies), following the manufacturer’s instructions. Isolated CD8^+^ T cells were cultured in RPMI-1640 Glutamax, supplemented with 10% FBS, 1% P/S and stimulated with anti-CD3/CD28-coated microbeads (Thermo Fisher Scientific) at a 2:1 bead: T cells ratio, in the presence of 50 IU/mL of human recombinant interleukin-2 (h-IL2; GlaxoSmithKline). Retroviral transduction of T cells was performed 48 hours postactivation. T cells were transferred in retronectin-coated plates (Takara) previously spinoculated with predetermined concentrations of retroviral particles at 2000×g for 1.5 hours. T cells were removed from retronectin the following day, and medium was refreshed every other day. CD3/CD28 beads were removed 5 days postactivation, and the T cells were subsequently maintained in medium supplemented with 10 ng/mL human IL-7 (Miltenyi) and 10 ng/mL IL-15 (Miltenyi) at 0.5–1×10^6^ T cells/mL until use. Phenotypic analysis was performed after day 7 to determine transduction efficiency and cells were used after day 9 for all assays.

### Murine T-cell isolation, stimulation, viral transduction, and expansion

Primary mouse T cells were isolated from the spleens of CD45.2^+^ HLA-A2^+^ mice, using a mouse CD8^+^ T cell isolation kit (StemCell Technologies) following the manufacturer’s instructions. Isolated T cells were stimulated with anti-CD3/CD28 beads (Thermo Fisher Scientific) at a 2:1 bead: T cells ratio in the presence of 50 IU/mL human IL-2 (GlaxoSmithKline). Retroviral transduction of T cells was performed 24 hours postactivation as previously described.^40^ Briefly, murine T cells were transferred to retronectin-coated (Takara) plates previously spinoculated with predetermined amounts of retroviral particles at 2000×g for 1.5 hours. T cells were removed from retronectin-coated plates the next day and maintained thereafter in complete medium supplemented with 10% heat-inactivated FCS, 1% P/S, 1 mM sodium pyruvate, 50 µM β-mercaptoethanol, 10 mM nonessential amino acids, 10 ng/mL human IL-7 (Miltenyi) and 10 ng/mL IL-15 (Miltenyi). CD3/CD28 beads were removed 7 days postactivation, and the T cells were cultured at 0.5–1×10^6^ T cells/mL until use. Phenotypic analysis of the cells for transduction efficiency was performed after day 7 of culture and cells were used after day 9 for all in vitro and in vivo assays.

### Flow cytometry

For flow cytometric analysis, single cell suspensions were stained with antibodies against human CD8 (RPA-T8) and β2m (2M2), and against mouse, CD11c (N418), Ly6G (1A8), Ly6C (HK1.4), F4-80 (ΒΜ8), MHC-II (M5/114.15.2), CD45.1 (A20), CD45.2 (104), CD11b (M1/70), CD64 (X54-5/7.1), CD19 (1D3), CD3 (17A2, 145-2 C11), CD4 (GK1.5), CD8 (5H10, 53.6.7), CD161 (PK136), CD69 (H1.2F3), CD137 (17B5), PD-1 (29F-1A12), CD25 (PC61.5), TIGIT (1G9), TIM-3 (RMT3-23). Antibodies were purchased from BD Biosciences, Biolegend, Thermo Fisher Scientific, and Beckman Coulter, or produced in-house from hybridomas at the flow cytometry platform of the University of Lausanne. A detailed list of differently labeled antibodies used in this study is found in online supplemental table 1. Expression of TCRs on the surface of transduced T-cells was detected either by specific phycoerythrin conjugated multimers from the Tetramer Core Facility at the University of Lausanne, or by staining with an anti-human TCR mAb, specific for the Vβ13.1 chain of the NY-ESO-1 TCR (IMMU 222, Beckman Coulter). Cell staining was performed at 4°C for 30 min, with prior blocking of mouse Fc receptors (Biolegend), when necessary. For detection of intracellular granzyme B, cells were fixed and permeabilized with the FoxP3 transcription factor staining buffer set (Thermo Fisher Scientific) at RT for 1 hour, and subsequently stained with anti-human/mouse Granzyme B at RT for 1 hour (GB11, Biolegend). DAPI (Sigma) or Live/Dead fixable Aqua Dead (Thermo Fisher Scientific) cell staining was used to exclude dead cells, according to the manufacturer’s instructions. Apoptotic cells were excluded by staining with Annexin V (BD Biosciences) at 4°C in the dark for 15 min. Cell acquisition was performed on an LSRII, LSR-SORP, or LSR-Fortessa flow cytometer (BD Biosciences) using DIVA software and data were analyzed using FlowJo (TreeStar).

### Cytokine production and T cell cytotoxicity assay by flow cytometry for human cells

In experiments with human T cells/tumor cells, 5×10^4^ rested NY-TCR^+^ T cells were co-cultured with 10^5^ NucLight Red^+^ tumor cells in complete RPMI-Glutamax medium for 24 hours. T cell numbers were normalized based on transduction efficiency and UTD cells were added as needed to ensure similar cell densities among the different conditions. Interferon-gamma (INF-γ) levels in collected cell-free supernatants were determined by Cytokine Bead Array (CBA; BD Biosciences) following the manufacturer’s protocol. T cell cytotoxicity was determined by flow cytometry analysis of the cells and was defined as the percentage of Annexin V^+^/DAPI^+^ tumor cells. Results were normalized to the percentage of AnnexinV^+^/DAPI^+^ in cultures of tumor cells alone.

### IncuCyte assay for imaging target cell killing

For cytotoxicity assays involving mouse cells, 1.5×10^4^ or 3×10^4^ rested NY-TCR^+^ T cells were co-cultured with 1.5×10^4^ tumor cells (pulsed or not with the NY peptide SLLMWITQA; C9A is to avoid disulfide bridge formation) in complete T cell medium for up to 96 hours, as indicated in figure legends. IncuCyte Cytotox Red reagent (Essen Biosciences) was added to assess cell death according to the manufacturer’s instructions. Internal experimental negative controls were included in all assays, including T cells alone and tumor cells alone to monitor spontaneous cell death over time. As an internal positive control, tumor cells alone were treated with 1% triton-X in PBS to evaluate maximal killing in the assay. Phase and red fluorescence images were acquired every 2 hours using IncuCyte S3 (Essen Biosciences). Tumor cell death was determined by following total red object area/mm^2^ over time.

### Cytokine production measurement assay in mouse T cells

For cytokine production assessment, 5×10^4^ rested NY-TCR^+^ mouse T cells were co-cultured with 5×10^4^ B16-A2Kb:NY or B16-A2Kb:MelanA cells in mouse T cell medium for 24 hours. T cell numbers were normalized based on transduction efficiency and UTD cells were added as needed to ensure similar cell densities among the different conditions. Cytokine and chemokine levels in collected cell-free supernatants were determined by CBA (BD Biosciences) following manufacturer’s instructions. Data were acquired on a CytoFLEX S cytometer (Beckman-Coulter) and analyzed with FCAP Array software (BD Bioscience).

### Adoptive TCR-T cell transfer

For xenograft studies, female NSG mice aged 8–12 weeks were subcutaneously inoculated on the flank with 10^6^ A375 melanoma cells. Male NSG mice 8–12 weekd were subcutaneously inoculated on the flank with 5×10^6^ Me275 cells. Concurrently, human T cells were activated, transduced, and expanded as described above. T cells were adoptively transferred to mice when tumors reached 50–100 mm^3^. Mice were treated twice with 10^7^ TCR-expressing T cells or equivalent number of UTD cells, with the second ACT performed 2–3 days after the first one. In syngeneic mouse studies, female CD45.1^+^ A2Kb transgenic C57BL/6 mice aged 6–12 weeks were subcutaneously inoculated on the flank with 2×10^5^ B16-A2Kb:NY or B16-A2Kb:MelanA melanoma cells. Concurrently, mouse T cells were activated, transduced, and expanded as described above. T cells were intravenously transferred to mice when tumors reached 50–100 mm^3^. Mice were treated twice with 10^7^ TCR-T cells, or equivalent number of UTD with the second ACT performed 2–3 days after the first one. Tumor growth was monitored by caliper measurements 2–3 times/week and tumor volume was calculated using the formula volume=½ (length×width^2^). Mice were sacrificed when tumors reached 1000 mm^3^, lost >20% of original weight, or became weak and moribund. Each group consisted of ≥5 mice.

### Ex vivo studies

To characterize in vivo responses to treatment, mouse tissues were collected at endpoint as indicated. Tumors were excised, weighed before dissociation, minced using a scalpel, and enzymatically dissociated in Liberase (Roche) and DNAseI (Roche) at 37°C for 1 hour. Single-cell suspensions were prepared by mechanical dissociation over a 70 mm strainer (Greiner). For characterizing murine immune infiltrate, including macrophages (CD3^−^CD19^−^Ly6C^−^MHC-II^+^CD11c^+^F4/80^+^CD64^+^), neutrophils (CD3^-^CD19^-^Ly6G^+^), dendritic cells (CD3^−^CD19^−^Ly6C^−^MHC-II^+^CD11c^+^F4/80^−^CD64^−^CD11b^+^), T cells (CD3^+^) and NK cells (CD3^−^CD19^−^CD161^+^) we used markers described by Lai *et al*.^82^

### Statistics

All statistical analyses were performed on GraphPad Prism V.6 software. Statistical tests used for each figure are described in the corresponding figure legend. P values <0.05 were considered statistically significant. Mean±SD was used to summarize the data, unless noted otherwise. Statistical differences in means of two groups were calculated by two-tailed parametric Student’s t-tests for unpaired data. Statistical comparisons in means of three groups or more were performed by one-way analysis of variance (ANOVA) or two-way ANOVA with correction for multiple comparisons using Tukey’s test (all groups compared) or Sidak’s test (two select groups compared). The Kaplan-Meier method was used to generate median survival, which was statistically analyzed by log-rank test. No statistical tests were used to predetermine sample sizes. Experiments in mice were performed with 5–10 mice per group as indicated in the figure legends based on previous experiments showing that this size could guarantee reproducibility and statistically significant differences. Tumor-bearing mice were assigned into treatment groups before T-cell infusion having similar mean tumor volumes and SD. To prevent cage effect in tumor growth, mice assigned the same treatment group were not rehoused together. Mice were treated by an operator who was blinded to treatment groups. All in vitro experiments were performed with T cells from a minimum of three independent healthy donors. The number of repetitions is indicated in the figure legends. All analyses of in vitro and in vivo data were based on objectively measurable data.

## supplementary material

10.1136/jitc-2024-009504 online supplemental file 1

## Data Availability

Data sharing is not applicable as no datasets were generated and/or analyzed for this study. Data are available on reasonable request. All data relevant to the study are included in the article or uploaded as online supplemental information.

## References

[1] Simoni Y, Becht E, Fehlings M (2018). Bystander CD8^+^ T cells are abundant and phenotypically distinct in human tumour infiltrates. Nature New Biol.

[2] Oliveira G, Stromhaug K, Klaeger S (2021). Phenotype, specificity and avidity of antitumour CD8^+^ T cells in melanoma. Nature New Biol.

[3] Stevanović S, Pasetto A, Helman SR (2017). Landscape of immunogenic tumor antigens in successful immunotherapy of virally induced epithelial cancer. Science.

[4] Arnaud M, Chiffelle J, Genolet R (2022). Sensitive identification of neoantigens and cognate TCRs in human solid tumors. Nat Biotechnol.

[5] Rosenberg SA, Packard BS, Aebersold PM (1988). Use of tumor-infiltrating lymphocytes and interleukin-2 in the immunotherapy of patients with metastatic melanoma. A preliminary report. *N Engl J Med*.

[6] Rosenberg SA, Yang JC, Sherry RM (2011). Durable complete responses in heavily pretreated patients with metastatic melanoma using T-cell transfer immunotherapy. Clin Cancer Res.

[7] Dafni U, Michielin O, Lluesma SM (2019). Efficacy of adoptive therapy with tumor-infiltrating lymphocytes and recombinant interleukin-2 in advanced cutaneous melanoma: a systematic review and meta-analysis. Ann Oncol.

[8] Besser MJ, Shapira-Frommer R, Itzhaki O (2013). Adoptive transfer of tumor-infiltrating lymphocytes in patients with metastatic melanoma: intent-to-treat analysis and efficacy after failure to prior immunotherapies. Clin Cancer Res.

[9] Zacharakis N, Chinnasamy H, Black M (2018). Immune recognition of somatic mutations leading to complete durable regression in metastatic breast cancer. Nat Med.

[10] Creelan BC, Wang C, Teer JK (2021). Tumor-infiltrating lymphocyte treatment for anti-PD-1-resistant metastatic lung cancer: a phase 1 trial. Nat Med.

[11] Tran E, Turcotte S, Gros A (2014). Cancer immunotherapy based on mutation-specific CD4+ T cells in a patient with epithelial cancer. Science.

[12] Deniger DC, Pasetto A, Robbins PF (2018). T-cell Responses to *TP53* 'Hotspot' Mutations and Unique Neoantigens Expressed by Human Ovarian Cancers. Clin Cancer Res.

[13] Stevanović S, Draper LM, Langhan MM (2015). Complete regression of metastatic cervical cancer after treatment with human papillomavirus-targeted tumor-infiltrating T cells. J Clin Oncol.

[14] Mullard A (2024). Tumour-infiltrating lymphocyte cancer therapy nears FDA finish line. Nat Rev Drug Discov.

[15] Thommen DS, Schumacher TN (2018). T Cell Dysfunction in Cancer. Cancer Cell.

[16] Aleksic M, Liddy N, Molloy PE (2012). Different affinity windows for virus and cancer-specific T-cell receptors: implications for therapeutic strategies. Eur J Immunol.

[17] Klebanoff CA, Chandran SS, Baker BM (2023). T cell receptor therapeutics: immunological targeting of the intracellular cancer proteome. Nat Rev Drug Discov.

[18] D’Angelo SP, Araujo DM, Abdul Razak AR (2024). Afamitresgene autoleucel for advanced synovial sarcoma and myxoid round cell liposarcoma (SPEARHEAD-1): an international, open-label, phase 2 trial. Lancet.

[19] Mullard A (2024). FDA approves first TCR-engineered T cell therapy, for rare soft-tissue cancer. Nat Rev Drug Discov.

[20] Reichenbach P, Giordano Attianese GMP, Ouchen K (2023). A lentiviral vector for the production of T cells with an inducible transgene and a constitutively expressed tumour-targeting receptor. Nat Biomed Eng.

[21] Schober K, Müller TR, Gökmen F (2019). Orthotopic replacement of T-cell receptor α- and β-chains with preservation of near-physiological T-cell function. Nat Biomed Eng.

[22] Chiesa R, Georgiadis C, Syed F (2023). Base-Edited CAR7 T Cells for Relapsed T-Cell Acute Lymphoblastic Leukemia. N Engl J Med.

[23] Courtney AH, Lo WL, Weiss A (2018). TCR Signaling: Mechanisms of Initiation and Propagation. Trends Biochem Sci.

[24] Stinchcombe JC, Asano Y, Kaufman CJG (2023). Ectocytosis renders T cell receptor signaling self-limiting at the immune synapse. Science.

[25] Giordano Attianese GMP, Ash S, Irving M (2023). Coengineering specificity, safety, and function into T cells for cancer immunotherapy. Immunol Rev.

[26] Lai JP, Robbins PF, Raffeld M (2012). NY-ESO-1 expression in synovial sarcoma and other mesenchymal tumors: significance for NY-ESO-1-based targeted therapy and differential diagnosis. Mod Pathol.

[27] Odunsi K, Jungbluth AA, Stockert E (2003). NY-ESO-1 and LAGE-1 cancer-testis antigens are potential targets for immunotherapy in epithelial ovarian cancer. Cancer Res.

[28] Chen YT, Scanlan MJ, Sahin U (1997). A testicular antigen aberrantly expressed in human cancers detected by autologous antibody screening. Proc Natl Acad Sci U S A.

[29] Jungbluth AA, Chen YT, Stockert E (2001). Immunohistochemical analysis of NY-ESO-1 antigen expression in normal and malignant human tissues. Int J Cancer.

[30] Gyurdieva A, Zajic S, Chang YF (2022). Biomarker correlates with response to NY-ESO-1 TCR T cells in patients with synovial sarcoma. Nat Commun.

[31] Zoete V, Irving M, Ferber M (2013). Rational Design of T Cell Receptors. Front Immunol.

[32] Zoete V, Irving MB, Michielin O (2010). MM-GBSA binding free energy decomposition and T cell receptor engineering. J Mol Recognit.

[33] Chen J-L, Stewart-Jones G, Bossi G (2005). Structural and kinetic basis for heightened immunogenicity of T cell vaccines. J Exp Med.

[34] Irving M, Zoete V, Hebeisen M (2012). Interplay between T cell receptor binding kinetics and the level of cognate peptide presented by major histocompatibility complexes governs CD8+ T cell responsiveness. J Biol Chem.

[35] Schmid DA, Irving MB, Posevitz V (2010). Evidence for a TCR affinity threshold delimiting maximal CD8 T cell function. J Immunol.

[36] Schmidt J, Chiffelle J, Perez MAS (2023). Neoantigen-specific CD8 T cells with high structural avidity preferentially reside in and eliminate tumors. Nat Commun.

[37] Ishihara M, Nishida Y, Kitano S (2023). A phase 1 trial of NY-ESO-1-specific TCR-engineered T-cell therapy combined with a lymph node-targeting nanoparticulate peptide vaccine for the treatment of advanced soft tissue sarcoma. Int J Cancer.

[38] Herrera FG, Ronet C, Ochoa de Olza M (2022). Low-Dose Radiotherapy Reverses Tumor Immune Desertification and Resistance to Immunotherapy. Cancer Discov.

[39] Lanitis E, Dangaj D, Irving M (2017). Mechanisms regulating T-cell infiltration and activity in solid tumors. *Ann Oncol*.

[40] Lanitis E, Rota G, Kosti P (2021). Optimized gene engineering of murine CAR-T cells reveals the beneficial effects of IL-15 coexpression. J Exp Med.

[41] Corria-Osorio J, Carmona SJ, Stefanidis E (2023). Orthogonal cytokine engineering enables novel synthetic effector states escaping canonical exhaustion in tumor-rejecting CD8+ T cells. Nat Immunol.

[42] Cohen CJ, Zhao Y, Zheng Z (2006). Enhanced Antitumor Activity of Murine-Human Hybrid T-Cell Receptor (TCR) in Human Lymphocytes Is Associated with Improved Pairing and TCR/CD3 Stability. Cancer Res.

[43] Dunn SM, Rizkallah PJ, Baston E (2006). Directed evolution of human T cell receptor CDR2 residues by phage display dramatically enhances affinity for cognate peptide-MHC without increasing apparent cross-reactivity. Protein Sci.

[44] Valitutti S, Müller S, Cella M (1995). Serial triggering of many T-cell receptors by a few peptide-MHC complexes. Nature New Biol.

[45] Vitiello A, Marchesini D, Furze J (1991). Analysis of the HLA-restricted influenza-specific cytotoxic T lymphocyte response in transgenic mice carrying a chimeric human-mouse class I major histocompatibility complex. J Exp Med.

[46] Men Y, Miconnet I, Valmori D (1999). Assessment of immunogenicity of human Melan-A peptide analogues in HLA-A*0201/Kb transgenic mice. J Immunol.

[47] Romero P, Valmori D, Pittet MJ (2002). Antigenicity and immunogenicity of Melan-A/MART-1 derived peptides as targets for tumor reactive CTL in human melanoma. Immunol Rev.

[48] Garcia Casado J, Janda J, Wei J (2008). Lentivector immunization induces tumor antigen‐specific B and T cell responses *in vivo*. Eur J Immunol.

[49] Valmori D, Gileadi U, Servis C (1999). Modulation of proteasomal activity required for the generation of a cytotoxic T lymphocyte-defined peptide derived from the tumor antigen MAGE-3. J Exp Med.

[50] Chapatte L, Colombetti S, Cerottini J-C (2006). Efficient Induction of Tumor Antigen–Specific CD8. Cancer Res.

[51] Yu YYL, Netuschil N, Lybarger L (2002). Cutting edge: single-chain trimers of MHC class I molecules form stable structures that potently stimulate antigen-specific T cells and B cells. *J Immunol*.

[52] Lybarger L, Yu YYL, Miley MJ (2003). Enhanced immune presentation of a single-chain major histocompatibility complex class I molecule engineered to optimize linkage of a C-terminally extended peptide. J Biol Chem.

[53] Truscott SM, Wang X, Lybarger L (2008). Human major histocompatibility complex (MHC) class I molecules with disulfide traps secure disease-related antigenic peptides and exclude competitor peptides. J Biol Chem.

[54] Barkal AA, Weiskopf K, Kao KS (2018). Engagement of MHC class I by the inhibitory receptor LILRB1 suppresses macrophages and is a target of cancer immunotherapy. Nat Immunol.

[55] Matsui M, Kawano M, Matsushita S (2014). Introduction of a point mutation into an HLA class I single-chain trimer induces enhancement of CTL priming and antitumor immunity. Mol Ther Methods Clin Dev.

[56] Yadav M, Jhunjhunwala S, Phung QT (2014). Predicting immunogenic tumour mutations by combining mass spectrometry and exome sequencing. Nature New Biol.

[57] Schrörs B, Hos BJ, Yildiz IG (2023). MC38 colorectal tumor cell lines from two different sources display substantial differences in transcriptome, mutanome and neoantigen expression. Front Immunol.

[58] Hebeisen M, Baitsch L, Presotto D (2013). SHP-1 phosphatase activity counteracts increased T cell receptor affinity. J Clin Invest.

[59] Cappell KM, Kochenderfer JN (2023). Long-term outcomes following CAR T cell therapy: what we know so far. Nat Rev Clin Oncol.

[60] Sykulev Y, Joo M, Vturina I (1996). Evidence that a single peptide-MHC complex on a target cell can elicit a cytolytic T cell response. Immunity.

[61] Stefanidis E, Semilietof A, Pujol J (2024). Combining SiRPα decoy-coengineered T cells and antibodies augments macrophage-mediated phagocytosis of tumor cells. J Clin Invest.

[62] Doran SL, Stevanović S, Adhikary S (2019). T-Cell Receptor Gene Therapy for Human Papillomavirus–Associated Epithelial Cancers: A First-in-Human, Phase I/II Study. JCO.

[63] Nagarsheth NB, Norberg SM, Sinkoe AL (2021). TCR-engineered T cells targeting E7 for patients with metastatic HPV-associated epithelial cancers. Nat Med.

[64] D’Angelo SP, Melchiori L, Merchant MS (2018). Antitumor Activity Associated with Prolonged Persistence of Adoptively Transferred NY-ESO-1 c259T Cells in Synovial Sarcoma. Cancer Discov.

[65] Vuillefroy de Silly R, Pericou L, Seijo B (2024). Acidity suppresses CD8 + T-cell function by perturbing IL-2, mTORC1, and c-Myc signaling. EMBO J.

[66] Cribioli E, Giordano Attianese GMP, Ginefra P (2022). Enforcing GLUT3 expression in CD8^+^ T cells improves fitness and tumor control by promoting glucose uptake and energy storage. Front Immunol.

[67] Ortiz-Miranda Y, Masid M, Jiménez-Luna C Transcriptional reprogramming by il-2 variant generates metabolically active stem-like t cells. Systems Biology.

[68] Ito M, Hiramatsu H, Kobayashi K (2002). NOD/SCID/gamma(c)(null) mouse: an excellent recipient mouse model for engraftment of human cells. Blood.

[69] Hu Z, Xia J, Fan W (2016). Human melanoma immunotherapy using tumor antigen-specific T cells generated in humanized mice. Oncotarget.

[70] Jespersen H, Lindberg MF, Donia M (2017). Clinical responses to adoptive T-cell transfer can be modeled in an autologous immune-humanized mouse model. Nat Commun.

[71] Norelli M, Camisa B, Barbiera G (2018). Monocyte-derived IL-1 and IL-6 are differentially required for cytokine-release syndrome and neurotoxicity due to CAR T cells. Nat Med.

[72] Rongvaux A, Willinger T, Martinek J (2014). Development and function of human innate immune cells in a humanized mouse model. Nat Biotechnol.

[73] Willinger T, Rongvaux A, Takizawa H (2011). Human IL-3/GM-CSF knock-in mice support human alveolar macrophage development and human immune responses in the lung. Proc Natl Acad Sci USA.

[74] Billerbeck E, Barry WT, Mu K (2011). Development of human CD4+FoxP3+ regulatory T cells in human stem cell factor-, granulocyte-macrophage colony-stimulating factor-, and interleukin-3-expressing NOD-SCID IL2Rγ(null) humanized mice. Blood.

[75] Zhou Q, Munger ME, Veenstra RG (2011). Coexpression of Tim-3 and PD-1 identifies a CD8+ T-cell exhaustion phenotype in mice with disseminated acute myelogenous leukemia. Blood.

[76] Shakiba M, Zumbo P, Espinosa-Carrasco G (2022). TCR signal strength defines distinct mechanisms of T cell dysfunction and cancer evasion. J Exp Med.

[77] Kotsiou E, Brzostek J, Gould KG (2011). Properties and Applications of Single-Chain Major Histocompatibility Complex Class I Molecules. Antioxidants & Redox Signaling.

[78] Webb B, Sali A (2016). Comparative Protein Structure Modeling Using MODELLER. Curr Protoc Bioinformatics.

[79] Rose PW, Prlić A, Altunkaya A (2017). The RCSB protein data bank: integrative view of protein, gene and 3D structural information. Nucleic Acids Res.

[80] Edgar RC (2004). MUSCLE: multiple sequence alignment with high accuracy and high throughput. Nucleic Acids Res.

[81] Pettersen EF, Goddard TD, Huang CC (2004). UCSF Chimera--a visualization system for exploratory research and analysis. J Comput Chem.

[82] Lai J, Mardiana S, House IG (2020). Adoptive cellular therapy with T cells expressing the dendritic cell growth factor Flt3L drives epitope spreading and antitumor immunity. Nat Immunol.

