# Supplementary material for: Preclinical model for evaluating human TCRs against chimeric syngeneic tumors
Source: J Immunother Cancer. 2024 Dec 22;12(12):e009504. doi: 10.1136/jitc-2024-009504 (PMC11667476; doi:10.1136/jitc-2024-009504)
Supplement: online supplemental file 1 [file jitc-12-12-s001.pdf]

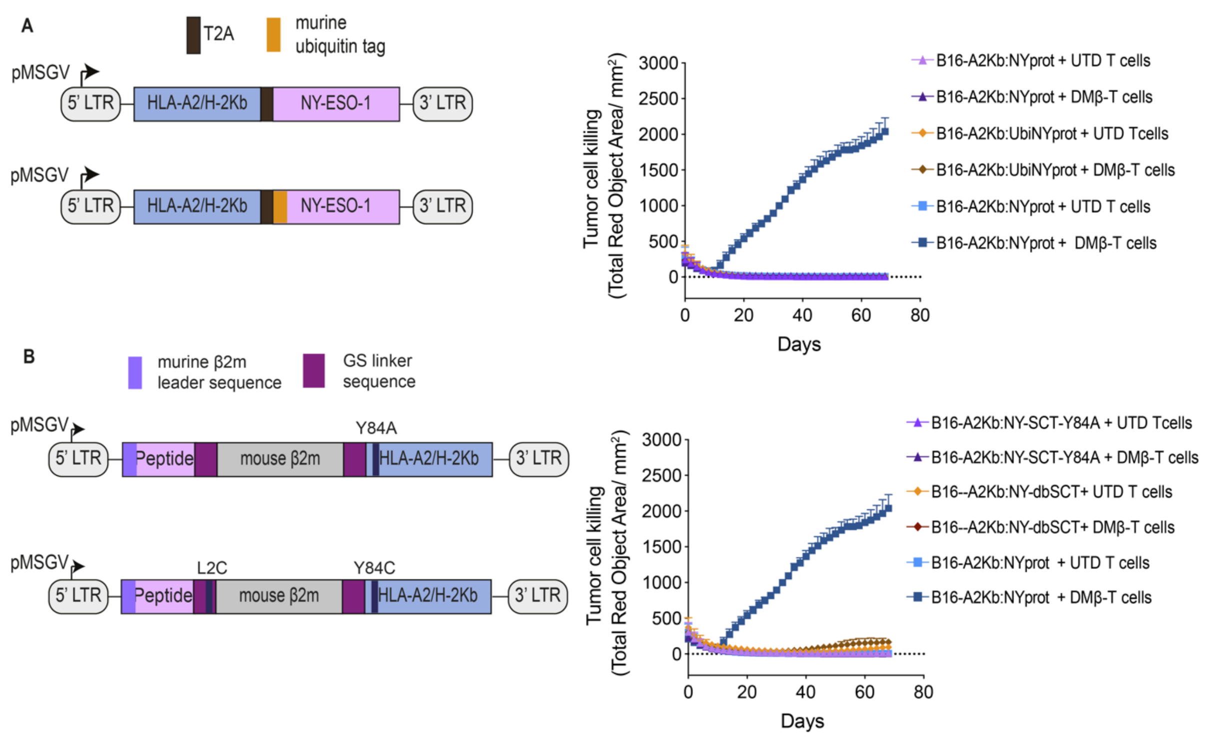

Supplemental Figure 1: Design strategies failing to enable B16 tumor cell expression of a chimeric HLA-A2/H-2Kb complex presenting the NY peptide. A) Left: Retroviral constructs encoding HLA-A2/H-2Kb (α1- α2 human, α3 and β2m of mouse origin) and the full human NY-ESO-1 sequence (B16-A2Kb:NYprot, top) or full human NY-ESO-1 sequence fused to a mouse ubiquitin tag (B16-A2Kb:UbiNYprot, bottom). **Right:** IncuCyte assay to evaluate killing of engineered B16 tumor cells by untransduced (UTD) and hybrid DMβ-T cells of mouse origin (n=3 donors). As a control, A2Kb^{+} B16 tumors cells were pulsed with NY-ESO-1 peptide (B16-A2Kb + NY pep). **B) Left:** Retroviral constructs encoding single chain trimers (SCT) comprising mouse β2m fused by flexible GS linkers to both the NY peptide and A2Kb (i.e., the α1- α2 peptide-binding groove of human origin, and α3 of mouse origin) further harboring mutation Y84A (in dark blue) in α1 previously described to stabilize peptide binding (top; B16-A2Kb:NY-Y84A) versus harboring a non-native disulfide-bridge (db) L2C and Y84C (in dark blue) previously described to improve complex stability (bottom; B16-A2Kb:dbNY). Right: IncuCyte assay to evaluate killing of SCT engineered B16 tumor cells by untransduced (UTD) and hybrid DMβ-T cells of mouse origin (n=3 donors). As a positive control, B16-A2Kb:NY cells (presented in Figure 3) were used.

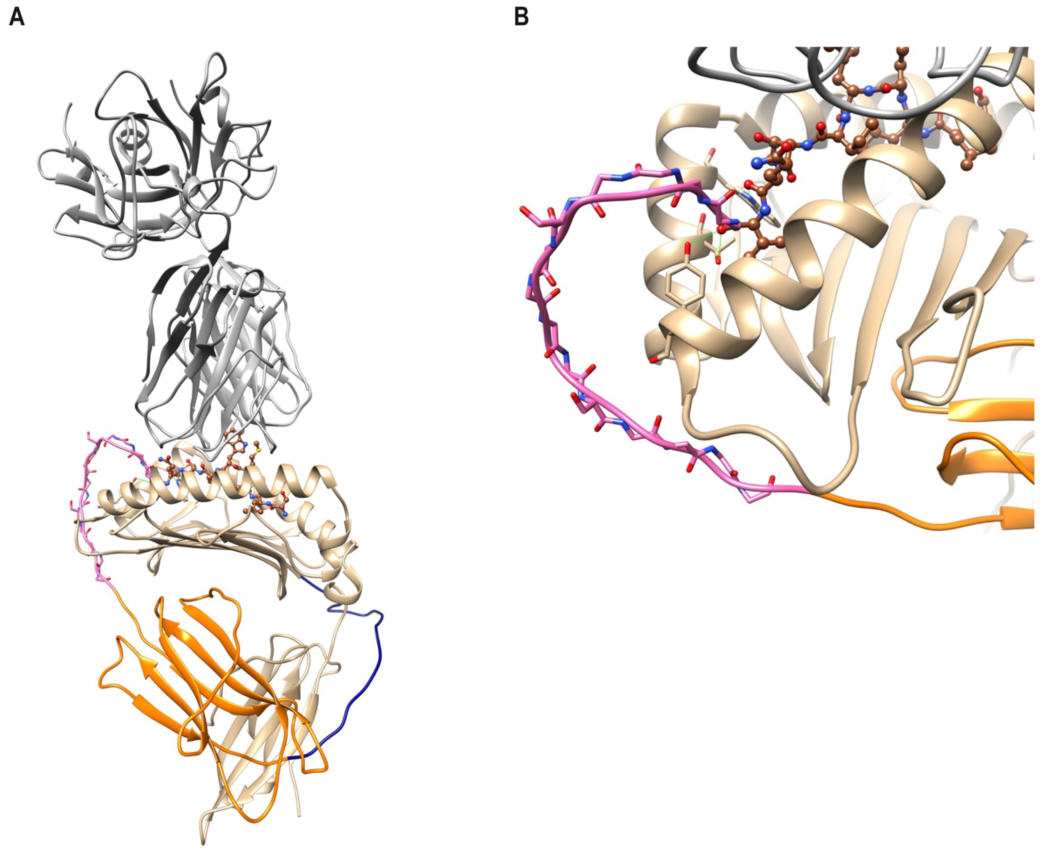

Supplemental Figure 2: Homology modeling of the HLA-A2/NY single chain trimer (SCT) in complex with the 1G4 TCR. A) 3D structural model of the complex between the 1G4 TCR (near identical in sequence to our A2/NY TCR) and the SCT comprising human β2m fused by GS linkers to both the NY peptide and to α1 comprising H74L described to stabilize peptide binding. The NY peptide is shown in ball and stick and colored in dark brown. The TCR, MHC and β2m are coloured in grey, light brown and orange, respectively. The GS linker between β2m and α1 is shown as a dark blue ribbon. The GS linker between β2m and the NY peptide is shown as a pink ribbon, with bonds displayed as thick lines. B) 3D structural model of the complex between the 1G4 TCR and the SCT. The NY peptide is shown in ball and stick and colored in dark brown. The TCR, MHC and β2m are coloured in grey, light brown and orange, respectively. The GS linker between β2m and the NY peptide is shown as a pink ribbon, with bonds displayed as thick lines. Hydrogen bonds are shown as thin green lines.

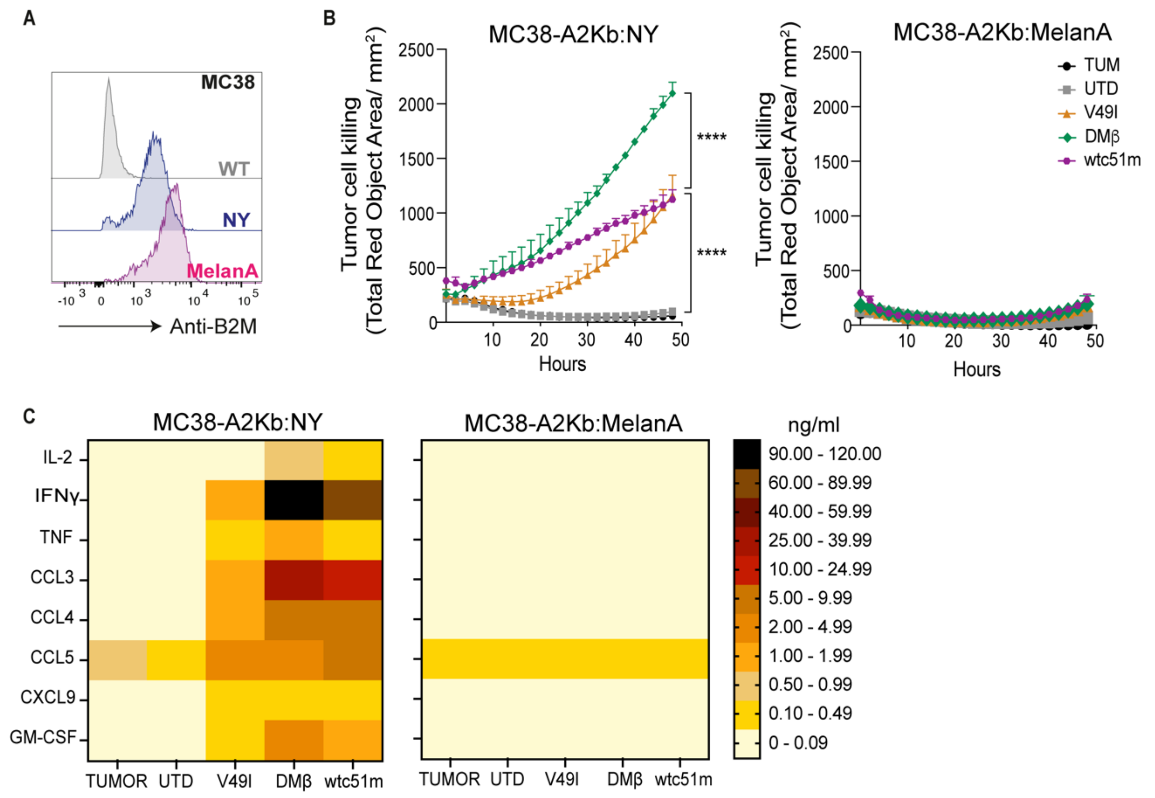

Supplemental Figure 3: A2/NY TCR-T cells are reactive against MC38 tumor cells engineered to express A2Kb:NY as a single chain trimer. A) Expression of the chimeric MHC molecule on the surface of mouse colorectal carcinoma MC38 cells detected by flow cytometry staining with an anti- human β2m antibody. **B)** Cytotoxicity of MC38-A2Kb:NY (left) and MC38-A2Kb:MelanA (right) cells by TCR modified T cells at E:T ratio = 1:1, followed by IncuCyte live cell imaging using Cytotox red dye (n=3 donors). **C)** Cytokine and chemokine secretion by TCR-T cells post 24h co-culture with MC38-A2Kb:NY and MC38-A2Kb:MelanA tumor cells at E:T ratio = 1:1 (n=3 donors) measured with CBA. Tumor cells alone were used as a control. Statistical analysis by two-way ANOVA (B), with correction for multiple comparisons by post hoc Tukey's test. ****P< 0.0001; ***P < 0.001; **P < 0.01; *P < 0.05.

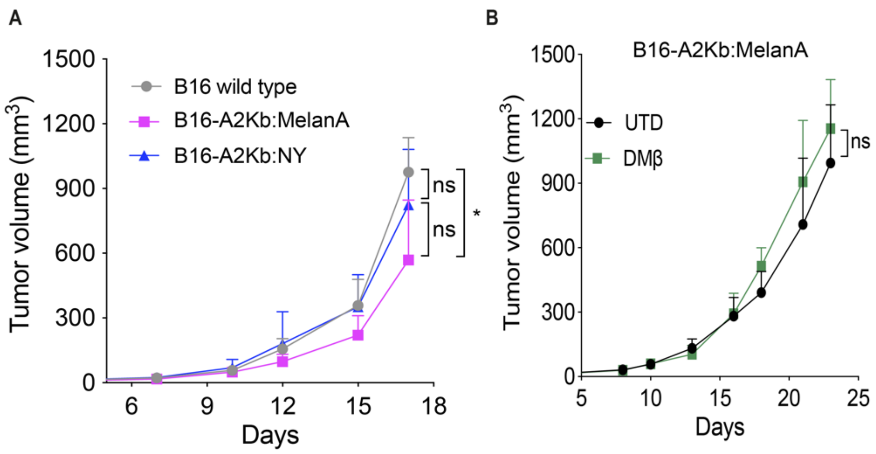

Supplemental Figure 4: Similar tumor growth curves for wild-type B16 versus gene-modified to overexpress the single chain trimers of A2Kb:NY and A2Kb:MelanA. A) Tumor growth curves of engineered B16-A2Kb:NY and B16-A2KB:MelanA cells compared to wild type B16 cells in HLA-A2/H-2Kb transgenic mice (n≥7 mice/ group, data representative of 2 independent experiments). **B)** Tumor growth curves of B16-A2Kb:MelanA cells following adoptive cell transfer of 10 million untransduced or DMβ- T cells. Cell numbers were corrected based on transduction efficiency, to ensure similar cell densities transferred in mice (n=6 mice/ group, data representative of 2 independent experiments). Statistical analysis by two-way ANOVA (A-B) with correction for multiple comparisons by post hoc Tukey's test. ****P< 0.0001; ***P < 0.001; **P < 0.01; *P < 0.05.

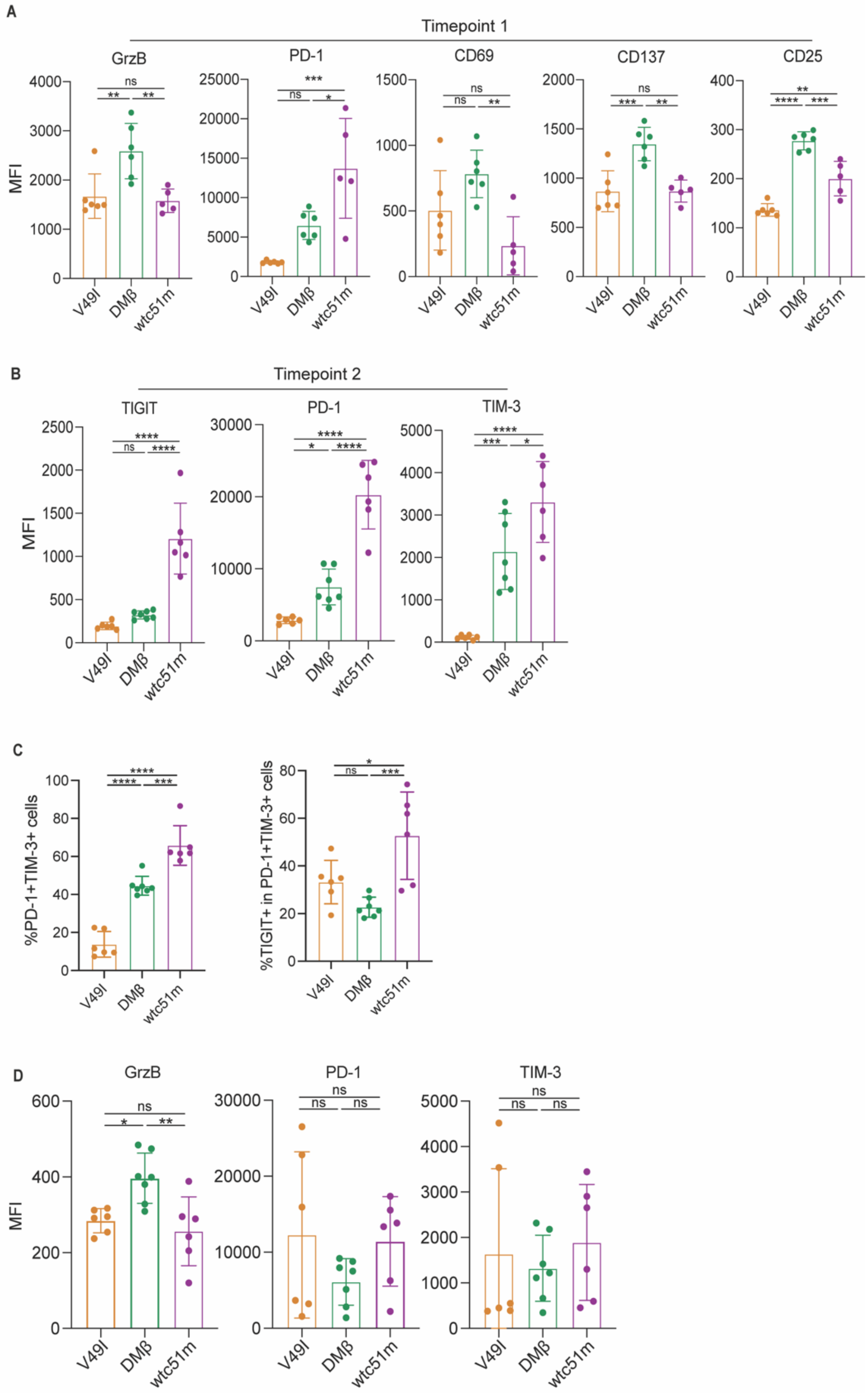

Supplemental Figure 5: Hybrid DMβ-T cells exhibit a superior fitness profile to V49I- and wtc51m-T cells post-transfer. A) Phenotypic analysis of markers for function, inhibition, and activation of adoptively transferred intratumoral CD45.2$^+$CD8$^+$ TCR-T cells shown as mean fluorescence intensity (MFI) 3 days post-ACT (n≥5 mice/ group). **B)** Expression levels (MFI) of inhibitory receptors on the surface of intratumoral CD45.2$^+$CD8$^+$ TCR-T cells 6 days

post-ACT (n=6 mice/ group). **C)** Left: Percentage of PD-1$^+$TIM-3$^+$ intratumoral CD45.2$^+$CD8$^+$ T cells 6 days post-ACT. Right: Percentage of TIGIT$^+$ in PD-1$^+$TIM-3$^+$ intratumoral CD45.2$^+$CD8$^+$ T cells 6 days post-ACT. **D)** Intracellular Granzyme B (GrzB) staining and cell surface expression of inhibitory receptors PD-1 and TIM-3 on endogenous CD45.1$^+$CD8$^+$ tumor infiltrating T cells 6 days post-ACT of TCR-T cells in B16-A2Kb:NY tumor-bearing mice. **A-D**) Representative data of two independent experiments. Statistical analysis by one-way ANOVA with correction for multiple comparisons by post hoc Tukey's test. ****P< 0.0001; ***P < 0.001; **P < 0.01; *P < 0.05.

Supplemental Table 1. List of antibodies used for flow cytometric experiments.

Antibody	Clone	Source	Identifier	Volume per 100µl or working concentration
Human CD8-Brilliant Violet 650 (BV650)	RPA-T8	Biolegend	#301042	0.5ul
Human β2-microglobulin	2M2	Biolegend	#316312	1ul
Human TCR Vbeta13.1-Phycoerythrin (PE)	IMMU 222	Beckman Coulter	#IM2292	7ul in cell pellet
Human/Mouse Granzyme B	GB11	Biolegend	#515406	1ul
Mouse CD3-Brilliant Violet 605 (BV605)	17A2	Biolegend	#100237	3ul
Mouse CD3-Phycoerythrin-Cyanine 5 (PE-Cy5)	145-2C11	Thermo Fisher Scientific	#15-0031-82	1ul
Mouse CD3- Alexa Fluor 700 (AF700)	17A2	Thermo Fisher Scientific	#56-0032-82	1ul
Mouse CD4- Brilliant Ultra Violet 395 (BUV395)	RM4-5	BD Bioscience	#563790	0.5ul
Mouse CD4-Phycoerythrin-Cyanine 5 (PE-Cy5)	GK1.5	Thermo Fisher Scientific	#15-0041-82	1ul
Mouse CD8-Phycoerythrin-Texas Red (PE-Tex Red)	5H10	Thermo Fisher Scientific	# MCD0817	0.5ul
Mouse CD8-Brilliant Violet 650 (BV650)	53.6.7	Biolegend	#100742	0.5ul

Mouse CD8-Phycoerythrin-Cyanine 5 (PE-Cy5)	53.6.7	Thermo Fisher Scientific	#14-0081-82	1ul
CD11b-Alexa Fluor 700 (AF700)	M1/70	Thermo Fisher Scientific	# 56-0112-82	0.5ul
Mouse CD11c-Brilliant Violet 605 (BV605)	N418	Biolegend	#117333	1ul
Mouse CD19-Phycoerythrin-Texas Red (PE-Tex Red)	1D3	Thermo Fisher Scientific	# RM7717	1ul
Mouse CD45.1-Allophycocyanin (APC)	A20	Thermo Fisher Scientific	#17-0453-82	1ul
Mouse CD45.1-Alexa Fluor 700 (AF700)	A20	Produced in-house		1ul
Mouse CD45.1-Fluorescein isothiocyanate (FITC)	A20	Thermo Fisher Scientific	#11-0453-82	0.5ul
Mouse CD45.2-Brilliant Violet 650 (BV650)	104	Biolegend	#109836	2ul
Mouse CD279 (PD-1)-Brilliant Violet 711 (BV711)	29F-1A12	Biolegend	#135231	1ul
Mouse CD366 (TIM-3)-Phycoerythrin (PE)	RMT3-23	Biolegend	#119704	1ul
Mouse CD64-Phycoerythrin (PE)	X54-5/7.1	Biolegend	#139304	1ul
Mouse F4/80-Allophycocyanin-Cyanine 7 (APC-Cy7)	BM8	Thermo Fisher Scientific	#47-4801-82	2ul
Mouse Ly6C-Fluorescein isothiocyanate (FITC)	HK1.4	Biolegend	#128006	1ul
Mouse Ly6G-Brilliant Violet 421 (BV421)	1A8	Biolegend	#127612	1ul
Mouse CD25-Allophycocyanin-Cyanine 7 (APC-Cy7)	PC61.5	Thermo Fisher Scientific	#47-0251-82	0.1ul
Mouse CD161 (NK1.1)-Phycoerythrin Cyanine 7 (PE-Cy7)	PK136	Thermo Fisher Scientific	#25-1619-42	2ul
Mouse CD69-Phycoerythrin (PE)	H1.2F3	Thermo Fisher Scientific	#12-0691-82	1ul
Mouse CD137 (4-1BB)-Peridinin chlorophyll protein-Cyanine5.5 (PerCP-Cy5.5)	17B5	Thermo Fisher Scientific	#46-1371-82	1ul

Mouse TIGIT-Phycoerythrin Cyanine 7 (PE-Cy7)	1G9	Biolegend	#142108	1ul
Mouse I-A/I-E (MHC-II)-Peridinin chlorophyll protein-Cyanine5.5 (PerCP-Cy5.5)	M5/114.15.2	Biolegend	#107626	1ul
Annexin V-V500		BD Biosciences	#561501	2.5ul
Multimer against HLA-A*0201/H-2Kb-NY-ESO-$1_{157-165}$ SLLMWITQC - Phycoerythrin (PE)		TCMetrix / Tetramer Core Facility in University of Lausanne		2ul in cell pellet
Human TruStain FcX™ (Fc Receptor Blocking Solution)		Biolegend	#422301	10ug/ml
Mouse TruStain FcX™ (Fc Receptor Blocking Solution)	2.4G2	Biolegend	# 101320	10ug/ml
Invitrogen™ LIVE/DEAD™ Fixable Aqua Dead Cell Stain Kit, for 405 nm excitation		ThermoFisher Scientific	#L34957	1/200
DAPI (4',6-Diamidino-2-Phenylindole, Dilactate)		Sigma-Aldrich	#D9542	100ng/ml
